# An observation-based scaling model for climate sensitivity estimates and global projections to 2100

**DOI:** 10.1007/s00382-020-05521-x

**Published:** 2020-12-18

**Authors:** Raphaël Hébert, Shaun Lovejoy, Bruno Tremblay

**Affiliations:** 1 Alfred-Wegener-Institut Helmholtz-Zentrum für Polar- und Meeresforschung, Telegrafenberg A45, Potsdam, 14473 Germany; 2 Department of Physics, McGill University, 3600 rue University, Montréal, Québec H3A 2T8 Canada; 3 Department of Atmospheric and Oceanic Sciences, McGill University, 845 rue Sherbrooke Ouest, Montréal, Québec H3A 0G4 Canada

**Keywords:** Global mean temperature, Projections, Climate sensitivity, RCP scenarios, Global warming, Scaling

## Abstract

We directly exploit the stochasticity of the internal variability, and the linearity of the forced response to make global temperature projections based on historical data and a Green’s function, or Climate Response Function (CRF). To make the problem tractable, we take advantage of the temporal scaling symmetry to define a scaling CRF characterized by the scaling exponent *H*, which controls the long-range memory of the climate, i.e. how fast the system tends toward a steady-state, and an inner scale $\tau \approx 2$   years below which the higher-frequency response is smoothed out. An aerosol scaling factor and a non-linear volcanic damping exponent were introduced to account for the large uncertainty in these forcings. We estimate the model and forcing parameters by Bayesian inference which allows us to analytically calculate the transient climate response and the equilibrium climate sensitivity as: $1.7^{+0.3} _{-0.2}$  K and $2.4^{+1.3} _{-0.6}$  K respectively (likely range). Projections to 2100 according to the RCP 2.6, 4.5 and 8.5 scenarios yield warmings with respect to 1880–1910 of: $1.5^{+0.4}_{-0.2}K$, $2.3^{+0.7}_{-0.5}$  K and $4.2^{+1.3}_{-0.9}$  K. These projection estimates are lower than the ones based on a Coupled Model Intercomparison Project phase 5 multi-model ensemble; more importantly, their uncertainties are smaller and only depend on historical temperature and forcing series. The key uncertainty is due to aerosol forcings; we find a modern (2005) forcing value of $[-1.0, -0.3]\, \,\,\mathrm{Wm} ^{-2}$ (90 % confidence interval) with median at $-0.7 \,\,\mathrm{Wm} ^{-2}$. Projecting to 2100, we find that to keep the warming below 1.5 K, future emissions must undergo cuts similar to RCP 2.6 for which the probability to remain under 1.5 K is 48 %. RCP 4.5 and RCP 8.5-like futures overshoot with very high probability.

## Introduction

The atmosphere is a complex system involving turbulent processes operating over a wide range of scales starting from millimeters at the Kolmogorov dissipation scale up to the size of the Earth, spanning over 10 orders of magnitudes in space. The dynamics are sensitive to initial conditions and there are deterministic predictability limits that are roughly equal to the eddy turn-over time (lifetime) of structures. For planetary scale structures in the atmosphere, the overall deterministic prediction limit of about 10 days corresponds to the scaling transition timescale $\tau _w$ from the *weather regime* to the *macroweather regime* (Lovejoy and Schertzer 2013a).

The atmospheric components of GCMs exhibit the same weather-macroweather scaling transition as the atmosphere and similar predictability limits. Beyond this horizon, the internal variability has to be interpreted stochastically so that a single GCM run is only one realization of the random process; at these timescales, weather models effectively become stochastic macroweather generators. For projections over multi-decadal timescales and beyond, multi-model ensembles (MME) that include several models are used. The mean of the MME is taken to obtain the deterministic forced component of temperature variability and average out the internal variability (Collins et al. 2013).

Emergent properties of the Earth’s climate, i.e. properties which are not specified a priori, are then inferred from GCM simulations. The *equilibrium climate sensitivity* (ECS) is such a property; it refers to the expected temperature change after an infinitely long time following a doubling in carbon dioxide ($\hbox {CO}_{2}$) atmospheric concentration. Another is the *transient climate response* (TCR), which is defined as the change in temperature after a gradual doubling of $\hbox {CO}_{2}$ atmospheric concentration over 70 years at a rate of 1% per year. However, it is not clear whether such emergent properties from computational models can be taken as genuine features of the natural world. The difficulty is that each GCM has its own climate (“structural uncertainty”) and this leads to very large discrepancies in ECS and TCR between GCMs; this underscores the need for qualitatively different approaches which can narrow down the properties of the real climate directly from observations.

The ecological consequences of global warming could be dire; therefore, better constraining climate sensitivity is of utmost importance in order to meet the urgency of adjusting economical and environmental policies. Since the United States National Academy of Sciences report (Charney et al. 1979), the likely range for the ECS has not changed and remains $[1.5 , 4.5] \, K$. A likely range corresponds to a 66% confidence interval (CI) and a very likely range corresponds to a 90% CI (Mastrandrea et al. 2010). In the Fourth Assessment Report (AR4) of the Intergovernmental Panel on Climate Change (IPCC), the lower limit was revised upward by 0.5 K, in accordance with the very likely range for ECS of GCMs from the Coupled Model Intercomparison Project phase 3 (CMIP3) ([2.1, 4.4] K). In the Fifth Assessment Report (AR5), the lower limit returned to that of AR3 and earlier assessment reports because of new observation-based results, while the very likely range of CMIP phase 5 (CMIP5) GCMs ([1.9, 4.5] K) remained very close to the CMIP3 one.

In this paper, we extend the approach of Hébert and Lovejoy (2018) to make climate projections through 2100. The approach is based on historical data and a simple model of the system memory based on scaling symmetries. The output of our model is then evaluated against the instrumental record using Bayes’ rules in order to obtain a probabilistic estimate of its parameters.

The paper is structured in 3 sections : methods and material, results and conclusion. The methods and material section is divided in three sub-sections. First, we introduce the linear response framework and then describe the scaling climate response function considered. Secondly, the radiative forcing, temperature and GCM simulations used will be described, and thirdly, we explain the method used for the estimation of the model parameters. The results section is also divided into three parts. The first sub-section presents the probability distribution functions for the parameters and applies them to decompose the anthropogenic and natural forced signals, and the internal variability. The second sub-section estimates the ECS and TCR with the parameters found, and the third uses the same parameters to produce global projections to 2100 which are better constrained than a 32 CMIP5 GCMs MME with which they are compared.

## Methods and material

### The linear response framework

The approach used in this study builds on the work of Hasselmann and other authors who worked within the linear response framework applied to the climate (Budyko, Sellers, Schwarz, Li and Jarvis, Held et al., Von Hateren, Rypdal and Rypdal, Dijkstra, Geoffroy et al., Marshall et al.). Below we first provide a review of this work for context. The reader solely interested in the current approach can jump to Sect. 2.2 without loss of continuity.

The internal components of the Earth system are often far from thermodynamic equilibrium, yet, taken as a whole, the Earth is not so far from an energy balance with outer space, and, at any moment, the difference between the incoming and outgoing energy fluxes is stored in the soil, ocean and atmosphere. The deviations from energy balance are typically small—at the level of a few percent—and this justifies the linear response framework. In this paper, we consider “zero-dimensional” energy balance models in which the Earth’s globally averaged temperature *T*(*t*) and forcing *F*(*t*) are anomaly time series, i.e. they represent deviations from their reference values.

The earliest linearized temperature response models were the Budyko-Sellers energy balance models based on the heat equation (Budyko 1969; Sellers 1969). These were originally one dimensional (zonally averaged) models, which, when globally averaged, are equivalent to the single “box” model (Hasselmann et al. 1993). Global energy balance box models are models of the temperature based on a homogeneous “box”. The box has a spatially uniform temperature that stores energy according to its heat capacity, density and size. If there is a single box, and one asssumes Newton’s law of cooling and that the heat crossing the surface is proportional to the first derivative of the order differential relationship with temperature, then, when perturbed, the Earth’s temperature will relax in an exponential way to its new steady-state temperature. When extra boxes are added, they mutually exchange heat, leading to a total response that is the sum of exponentials.

Hasselmann et al. (1993, 1997) already noted that it was desirable to use the more general linearized framework of response functions. This, they argued, was because empirical box models with a small number of degrees of freedom “lose the detailed information on the climate state and therefore cannot be readily constrained to conform to the detailed linearized dynamics of a more realistic CGCM climate model.” In this context, the response functions are called “climate response functions” (CRFs) and, following Hasselmann et al. (1993), we have a choice between the equivalent impulse CRFs $G_{\delta }(t)$ and step CRFs $G_{\varTheta }(t)$. The subscript “$\delta$” indicates Dirac delta function and “$\varTheta$” is for its integral, the step (Heaviside) function ($\varTheta =1$ for $t>0$ and $\varTheta = 0$ for $t \le 0$).

Hasselmann et al. (1993) and especially Hasselmann et al. (1997) already pointed out the advantages of the step CRF that relates the forcing and temperature via:

1 $$\begin{aligned} T(t)=s \int _{-\infty }^t G_{\varTheta } (t-t') F'(t')dt', \end{aligned}$$

where the $F'(t)$ is the time derivative of the forcing *F*(*t*) and $s$ is the equilibrium climate sensitivity (ECS) with units of $\mathrm{K}$ per doubling of $CO_2$ (see Eq. 15 below). While Hasselmann et al. (1993) incorporated the sensitivity into the definition of $G_{\varTheta }(t)$, writing the response with the separate factor $s$ has the advantage that $G_{\varTheta }(t)$ is dimensionless and $s$ and *F*(*t*) have their usual dimensions. For generality, we have also extended the range of integration to cover the entire past. In advocating Eq. 1, Hasselmann et al. (1997) pointed out that “The formulation of the climate response in terms of a response integral (i.e. step response) rather than in the traditional form of a differential equation for a box model has further advantages: it is not limited to simple low-order differential equations...”. Another advantage, later emphasized by Marshall et al. (2014, 2017), ) is that, physically, basing the theory on $G_{\varTheta }(t)$ is equivalent to studying the temperature response in classical $\hbox {CO}_{2}$ doubling experiments; other advantages are discussed below. The equivalence between the impulse and step CRFs arises because the step function $\varTheta (t)$ is the integral of $\delta (t)$ so that:

2 $$\begin{aligned} G_{\delta}(t)=G'_{\varTheta }(t) \end{aligned}$$

The temperature response in terms of $G_{\delta (t)}$ rather than $G_{\varTheta (t)}$ can thus easily be obtained by integrating Eq. 1 by parts to yield:

3 $$\begin{aligned} T(t)=s \int _{-\infty }^t G_{\delta } (t-t') F(t')dt' \end{aligned}$$

In the integration by parts, we used Eq. 2 and the boundary conditions $G_{\delta }(0)=0$ and $F(-\infty )=0$. Since causality requires $G_{\varTheta }(t)=G_{\delta }(t)=0$ when $t \le 0$, the former condition $G_{\delta }(0)=0$ is satisfied by physical systems. Similarly, the relation $F(-\infty )=0$ is not a restriction as it can be regarded simply as the definition of a convenient reference level of the forcing.

Unfortunately, without more assumptions or information, the linear framework of Eq. 1 (or Eq. 3) is unmanageably general. In order to make progress, Hasselmann et al. (1997) proposed a response function consisting of a sum of N exponentials - effectively an N box model (although without using differential equations: the boxes were only implicit). Nevertheless, they ultimately chose $N = 3$ out of practical necessity—so as to fit GCM outputs. Following the more usual procedure of deriving the impulse responses from linear differential equations (where impulse CRFs are called “Green’s functions”), Li and Jarvis (2009) used Laplace transforms to explicitly show that polynomial forcings of nth ordered differential equations (with constant coefficients and with n an integer), can quite generally be reduced to sums of exponentials. However, in the application part of their paper, they nevertheless used the value N = 3. The N exponential model was later advocated by van Hateren (2013), by the IPCC AR5 (2013, section 8.SM.11.2), and more recently by Frederiksen and Rypdal (2017). However, each exponential has its own amplitude and time constant so that even if we exclude the climate sensitivity parameter, the N exponential models have 2N parameters. This rapidly becomes an unmanageably large number so that in practice $N = 2$ or $N = 3$ are often chosen.

An interesting exception is van Hateren (2013) who used $N = 6$, but avoided fitting the implicit $2N = 12$ independent parameters by linking the amplitudes and time constants by a power law, calling the resulting four parameter model a “fractal climate response” model. His resulting step CRF model specifies four parameters: low and high frequency truncations, a logarithmic oscillation frequency and an overall scaling exponent. In the model we develop below, we eliminate the unnecessary oscillations and low frequency cutoff, thus reducing the step CRF to only two parameters. Using van Hateren’s empirically fitted parameters yields an impulse response that over the range of about 6 months to 1000 years is within a factor of $\approx$ 2 of our two-parameter model described below. For comparison, the key exponent *H* (denoted by q in his notation) was estimated as $-0.15$ compared to our value of $-0.5 ^{+0.4}_{-0.5}$ (below).

An approach related to the scaling CRF we develop below is that of Procyk et al. (2020) which is based on the Earth energy balance. A fractional generalization of conventional box models was considered and solved using Mittag–Leffler functions, often called generalized exponentials, also characterized by a scaling exponent *H* which was estimated as $H \in [0.33,0.44]$ and is equivalent to the negative of the SCRF’s H (below).

Although these authors proposed exponentials largely on mathematical grounds, the majority of linear response theory applications attempt to give physical interpretations of their parameters, especially their time constants, and these have not been very satisfactory. If each exponential can be modelled by a box that effectively stores heat, then it is not clear what the box should represent physically. If one chooses the atmosphere (e.g. Dijkstra 2013), then one obtains a short relaxation time $\tau$ of the order of days, whereas if one chooses the ocean, then a wide range of time scales can be obtained depending on the thickness of the relevant ocean layer.

Several estimates of the fast $\tau$, which would correspond to the rapidly equilibriating mixed-layer of the ocean, find values below 10 years: $\tau = 8.5 \pm 2.5$ years (Schwartz 2008), $\tau \approx 4$ years (Held et al. 2010), $\tau \in [1,6]$ years (Geoffroy et al. 2013), $\tau = 4.3 \pm 0.6$ years (Rypdal and Rypdal 2014). The estimates of the slow component, which would correspond to the deep ocean, are widely divergent and Geoffroy et al. (2013) concludes it should be between 60 and 700 years, an interval spanning an order of magnitude well above what can be directly probed from historical observations. The IPCC AR5 suggests a double exponential model with $\tau = 8.4$ years and $\tau = 409.5$ years.

But the box models are overly simple: in reality, the earth is highly heterogeneous with dynamical processes redistributing energy over a huge number of degrees of freedom, and a multitude of storage mechanisms, covering a wide ranges of scales. What we really need is a phenomenological model that is approximately valid for the globally averaged temperature, an equation that is valid at time scales longer than the weather scales (about 10 days). Once we accept that our equation is at best valid for averages (globally and over weather scales), it is no longer necessary to constrain the model to a single—or even a small number of degrees of freedom—slabs or boxes.

When *F*(*t*) is a step function, Eqs. 1 and 3 describe a linear system that is perturbed by a forcing and that subsequently relaxes to a new steady-state temperature. The problem is that the range of time scales over which the system reacts to a forcing is huge; indeed, due to scaling symmetries, as recognized by Rypdal (2012) and van Hateren (2013), the response is closer to a power law. We therefore seek to go beyond exponentials, while still restricting our attention to CRFs that correspond to processes which can relax to a stable state of energy balance. To see what constraints such “generalized” relaxation imposes, the step CRF is particularly convenient.

For example, for a physical system, a finite step forcing:

4 $$\begin{aligned} F(t)=F_0 \varTheta (t) {; }\,\, \, \, \varTheta (t)={\left\{ \begin{array}{ll} 1 \text { when }t\ge 0\\ 0 \text { when } t< 0\\ \end{array}\right. } \end{aligned}$$

must give a finite response. Since in Eq. 1 we included the extra sensitivity factor $s$, without loss of generality we can consider only normalized step CRF such that:

5 $$\begin{aligned} lim_{t \rightarrow \infty } G_{\varTheta }(t)=1 \end{aligned}$$

Since $s F_0$ is the new steady-state temperature, $s$ is the usual ECS. In addition, $G_{\varTheta }$ should be constrained to functions such that a steady state is established monotonically; we should exclude step responses that oscillate or that overshoot the steady state before returning to it:

6 $$\begin{aligned} G'_{\varTheta }(t)=G_{\delta }(t)>0 \end{aligned}$$

In addition, the response to a positive forcing should be positive so that combining these constraints, and using Eqs. 5, 6 and causality ($G_{\varTheta }(0) = 0$) we obtain :

7 $$\begin{aligned} 0 \le G_{\varTheta }(t) \le 1 \end{aligned}$$

Systems whose step CRFs respect Eqs. 6 and 7 thus define physically plausible generalized relaxation processes. As an example, the classical relaxation box/exponential model yields:

8 $$\begin{aligned} \begin{aligned} G_{\varTheta , \, box}(t)=1-e^\frac{-t}{\tau }{; } \,\, \, \, G_{\delta , \, box}(t)=\tau ^{-1}e^\frac{-t}{\tau } {; } \,\, \, \,&t>0 \\ G_{\varTheta , \, box}(t)=G_{\delta , \, box}(t)=0 {; } \,\, \, \,&t \le 0 \end{aligned} \end{aligned}$$

where $\tau$ is the “relaxation time”, the characteristic time associated with the return to a state of energy balance. We see that the approach to the steady-state is exponentially fast. Although the box model is usually specified via a differential equation, from the above, we see that it could equivalently be specified by $G_{\varTheta , \, box}(t)$; indeed, it is easy to verify that $G_{ \varTheta , \, box}(t)$ satisfies the standard box-model relaxation equation:

9 $$\begin{aligned} \tau \frac{d G_{ \varTheta , \, box}}{dt} +G_{ \varTheta , \, box}=\varTheta (t) \end{aligned}$$

Taking derivatives, we also confirm that $G_{ \delta , \, box}(t) = G'_{ \varTheta , \, box}(t)$ is indeed the impulse response for the operator.

Other simple CRF’s have been proposed, notably the Dirac function itself with a lag $t_0$:

10 $$\begin{aligned} G_{\varTheta }(t)=\varTheta (t -t_0) \text {; } \,\, \, \, G_{\delta }(t)=\delta (t-t_0) \end{aligned}$$

Which implies:

11 $$\begin{aligned} T(t)=s F(t-t_0) \end{aligned}$$

This has been used by Lean and Rind (2008) (with $t_0 = 10$ years as part of multiple regression), and directly by Lovejoy (2014) who varied $t_0$ in the range 0–20 years. In both cases, the forcing was at annual or longer temporal resolution so the response was not really instantaneous as Eq. 10 implies.

### Scaling relaxation processes

**Fig. 1:**
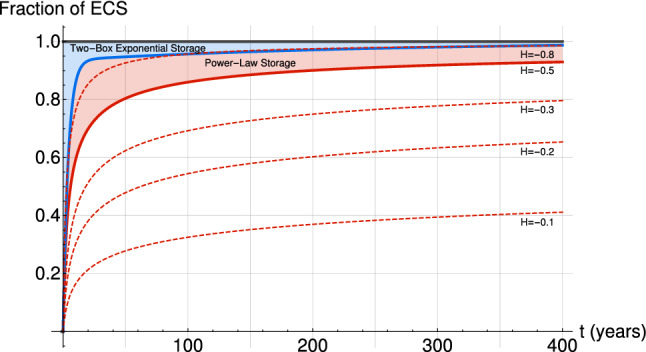
A nondimensional comparison of the step SCRF (Eq. 12, red) with the classical two-box exponential step CRF (Eq. 8, blue). The nondimensional (step) forcing (black) is also shown, and the difference between this forcing and the response is the rate of energy storage. The parameters for the two-box exponential are the best estimates from Geoffroy et al. (2013): $\tau _{fast}=4.1 \, years$, $\tau _{slow}=249 \, years$, $C=7.3 \, year \, W m^{-2} K^{-1}$ and $C_0=106 \, year \, W m^{-2} K^{-1}$ while for the SCRF we use $\tau =2 \, years$ and show the curves for different *H* values indicated on the graph

The Dirac CRF has essentially no memory, and the box model has an exponentially decaying one, neither is satisfactory, unless they are combined into a model with multiple response times. To be more realistic, the CRF should satisfy scaling symmetries (respected for example by the GCMs in control runs); we should therefore choose functions that correspond to scaling (power law) relaxation processes. The simplest scaling CRF (SCRF) that satisfies Eqs. 5 and 6 is:

12 $$\begin{aligned} G_{\varTheta }(t)={\left\{ \begin{array}{ll} 1- \left( 1+\frac{t}{\tau } \right) ^H \text { when } \,\, \, \, t \ge 0 \\ 0 \,\, \, \,\,\, \, \,\,\, \, \,\,\, \, \,\, \, \,\, \, \,\,\, \,\,\, \, \,\,\, \text { when } \,\, \, \, t<0 \end{array}\right. } \end{aligned}$$

where the requirement $H<0$ is needed so that $\lim _{t \rightarrow \infty } G_{\varTheta }(t)=1$ and the truncation at a small timescale $\tau$ is necessary so that $G_{\varTheta }(0)=0$. This step SCRF describes a power law relaxation process, thus converging more slowly, and realistically, to a steady-state than typical exponential models (Fig. 1 ), with scaling exponent *H* ($H<0$) and a corresponding impulse SCRF:

13 $$\begin{aligned} G_{\delta }(t)={\left\{ \begin{array}{ll} -\frac{H}{\tau } \left( 1+\frac{t}{\tau } \right) ^{H-1} \text { when } \,\, \, \, t \ge 0 \\ 0 \,\, \, \, \,\,\, \, \,\,\, \, \,\,\, \, \,\,\, \, \, \,\, \, \,\,\, \, \,\,\, \, \,\,\, \, \, \, \text { when } \,\, \, \, t<0\end{array}\right. } \end{aligned}$$

so that the impulse CRF is a also a truncated power law.

Rypdal (2012) already proposed a similar CRF with $H > 0$, which has the advantage of not needing the truncation $\tau$ at small time scales. This allows the modelling of the high-frequency with the same scaling by the simple addition of a random white noise forcing to the deterministic forcing. This came at the expense of divergence at large time scales, the *runaway Green’s function effect* (Hébert and Lovejoy 2015), since any finite increase in forcing would lead to an ever increasing temperature response, i.e. an infinite ECS. An interesting alternative to model high- and low-frequency regimes with scaling regimes can be obtained by the fractional generalization of the energy-balance equation, leading to a CRF with a similar (convergent) low-frequency behaviour as the SCRF (Procyk et al. 2020).

**Fig. 2:**
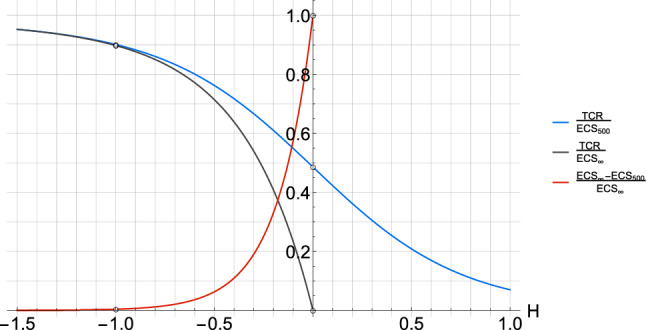
The analytical ratio between TCR and ECS is shown as a function of the scaling exponent *H* for a high frequency cutoff $\tau = 2 \, years$. The ratio of TCR to ECS$_{500}$ is also shown for equilibrium defined at $500 \, years$ (black) along with the true equilibrium at infinity (blue) and the leftover warming fraction (red) between 500 years and infinity

It is straightforward to analytically calculate the expected temperature increase using the truncated SCRF in Eq. 13 in response to specific forcing scenario such as to recover TCR and ECS (See Appendix A for details). With the same forcing scenario as ECS, we also define ECS$_{500}$ as the expected temperature 500 years after the $\hbox {CO}_{2}$ doubling rather than at infinity. This is a more relevant ECS measure from a human perspective and helps to illustrate the contribution to the ECS of the very long-memory beyond 500 years. The ratio of TCR to ECS (Eq. A10) changes from unity for $H \rightarrow -\infty$ to zero for $H \ge 0$ where ECS diverges while TCR remains finite; conversely, the fraction of warming left between 500 years and infinity ($(ECS-ECS_{500})ECS^{-1}$) goes from zero to unity when *H* goes from negative infinity to zero and greater (Fig. 2).

### Data

#### Radiative forcing data

In this paper, we consider three sources of external forcing: solar and volcanic which are natural, and anthropogenic forcing which involves several forcing agents produced by humans. The forcing is usually expressed in $\,\,\mathrm{W\, m}^{-2}$; however the climate sensitivity is commonly measured in K per doubling of $\hbox {CO}_{2}$. Therefore, it is convenient to also define forcing as a fraction of the forcing imparted by a doubling of $\hbox {CO}_{2}$ concentration: $\varDelta F_{2 \times \,\,\mathrm{CO}_2}$. The generally accepted (approximate) carbon dioxide concentration to forcing relationship is:

14 $$\begin{aligned} F_{\,\,\mathrm{CO}_2}(\rho )=3.71 \,\,\mathrm{W\, m}^{-2} \log _2 \frac{\rho }{\rho _0} \end{aligned}$$

where $F_{\,\,\mathrm{CO}_2}$ is the forcing due to carbon dioxide, $\rho$ is the carbon dioxide concentration and $\rho _0$ is its pre-industrial value which we take to be $277 \, ppm$. Therefore,

15 $$\begin{aligned} \varDelta F_{2 \times \,\,\mathrm{CO}_2} = F_{\,\,\mathrm{CO}_2} (2 \rho _0)=3.71 \,\,\mathrm{W\, m}^{-2} \end{aligned}$$

*a) Greenhouse Gas Forcing*

**Fig. 3:**
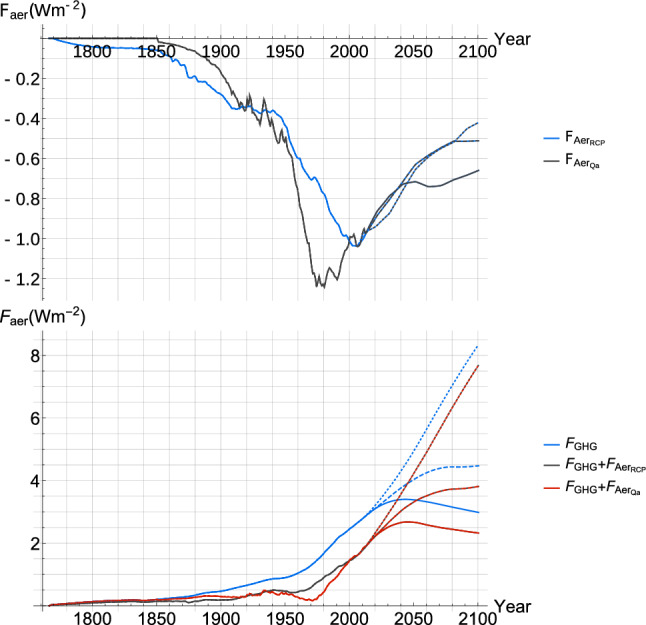
(top) The anthropogenic aerosol forcing series used, $F_{Aer_{RCP}}$ (blue) and $F_{Aer_{Q_a}}$ (black), are shown over the historical period and over the projection period until 2100 for RCP 2.6 (solid), RCP 4.5 (dashed), and RCP 8.5 (dotted); $F_{Aer_{Q_a}}$ was extended with the $F_{Aer_{RCP}}$ series. (bottom) The greenhouse gas forcing series $F_{GHG}$ (blue) and the total anthropogenic forcing series, adding $F_{Aer_{RCP}}$ (black) or $F_{Aer_{Q_a}}$ (red) to $F_{GHG}$, are shown over the historical period and projection period for the 3 RCP scenarios considered, as above

Anthropogenic influences on the climate have been recognized as the main driver of the global warming characteristic of the last century, and the related forcing is mostly due to historical changes in atmospheric composition. Future anthropogenic forcing is prescribed in four scenarios, the Representative Concentration Pathways (RCPs), established by the IPCC for CMIP5 simulations : RCP 2.6, RCP 4.5, RCP 6.0 and RCP 8.5 (Meinshausen et al. 2011), shown in Fig. 3. They are named according to the total radiative forcing in $\,\,\mathrm{Wm}^{-2}$ expected in the year 2100 and are motivated by complex economic projections, expected technological developments, and political decisions. The scenarios allow us to verify and compare results from our observations-based SCRF model with CMIP5 simulations. Generally, RCP 6.0 was left out of the analysis since fewer CMIP5 modeling groups performed the associated runs.

The measure of anthropogenic forcing $F_{Ant}$ used in this paper is the carbon dioxide equivalent $F_{{{\rm CO}_{2}}EQ}$ series given in the RCP scenarios. It corresponds to the combined effective radiative forcing produced by Long Lived Greenhouse Gases (GHG) $F_{GHG}$: carbon dioxide, methane, nitrous oxide and fluorinated gases, controlled under the Kyoto protocol, ozone depleting substances, controlled under the Montreal Protocol, and aerosols.

Chapter 8 of the IPCC AR5 reports the increase from 1750 to 2005 of effective radiative forcings with a very likely (90%) confidence interval (CI); we summarized them in Table 1 to evaluate the relative uncertainty of the different anthropogenic forcing agents. Note that we will report likely and very likely (symmetrical) CI at the 66% and 90% confidence level, respectively, throughout this work (i.e. $\pm 1$ and $\pm 1.645$ standard deviations respectively), in accordance with the IPCC. The largest forcing increase stems from GHG, in particular carbon dioxide, and it has a relatively small uncertainty.

*b) Aerosol forcing* There are also negative contributions to anthropogenic forcing from aerosols’ direct effect and indirect cloud albedo effects, both with very high relative uncertainties. The total anthropogenic change in effective radiative forcing is certainly positive, due to the strong GHG forcing, but the large uncertainty on aerosol forcing strongly dominates the total uncertainty. We therefore introduce the aerosol linear scaling factor $\alpha$ as an extra parameter to scale aerosol forcing (see Eq. 21 below).

The aerosol forcing in the RCP files $F_{Aer_{RCP}}$ is given implicitly; it can be obtained by subtracting the combined effective radiative forcing from gases controlled under the Kyoto protocol, $F_{Kyt}$, and from those controlled under the Montreal protocol, $F_{Mtl}$ (Fig. 3) from the ${\,\,\mathrm{CO}_2}_{EQ}$ forcing. $F_{Mtl}$ is given in CFC-12 equivalent concentration and we use the relation from Ramaswamy et al. (2001) to convert back to $\,\,\mathrm{W\, m}^{-2}$.

The very likely CI given for the modern value, defined in 2005, of total aerosol forcing in the IPCC AR5 is $[-1.9,-0.1] \,\,\mathrm{W\, m}^{-2}$, but Stevens (2015) (S15) demonstrates that a forcing more negative than $-1 \,\,\mathrm{W\, m}^{-2}$ is implausible and suggests, combined with results from Murphy et al. (2009) tightening the upper bound to $-0.3 \,\,\mathrm{W\, m}^{-2}$, that the interval be revised to $[-1.0,-0.3]\,\,\mathrm{W\, m}^{-2}$.

S15 proposes a three parameter model to derive the aerosol direct and indirect forcing directly from anthropogenic sulfur dioxide emissions $Q_a$ :

16 $$\begin{aligned} F_{Aer_{Q_a}}=\gamma Q_a + \beta \log \frac{Q_a}{\bar{Q_n}} \end{aligned}$$

where $Q_a$ is the annual anthropogenic sulfur dioxide emissions, $\gamma$ is the direct effect coefficient, $\beta$ is the indirect effect coefficient and $\bar{Q_n}$ is the mean natural sulfur dioxide atmospheric source strength. For a given set of parameters, we can obtain a forcing series which is highly correlated (r = 0.95) with $F_{Aer_{RCP}}$, and also with $Q_a$ (r = $-0.996$) itself since the log term is approximately linear near $\bar{Q_n}$. Therefore, we also include in the analysis an alternate aerosol forcing series for comparison which takes $Q_a$ as a linear surrogate for the total aerosol forcing such that :

17 $$\begin{aligned} F_{Aer_{Q_a}} \approx \gamma ^* Qa \end{aligned}$$

where the coefficient $\gamma ^*$ is taken to be equal to $-9.3 \times 10^{-6} \,\,\mathrm{W\, m}^{-2} Tg^{-1} yr$ so that the modern (2005) value of $F_{Aer_{Qa}}$ and $F_{Aer_{RCP}}$ are both about $-1.0 \,\,\mathrm{W\, m}^{-2}$. This method allows us to derive an aerosol forcing series (Fig. 3) directly from sulfur dioxide emission data that does not rely on GCMs. We decided to present as the main result estimates made using ${F_{Aer}}_{RCP}$ given it is based on a more accepted aerosol forcing series closer to that used in the MME we use for comparison, and results based on ${F_{Aer}}_{Qa}$ are presented alongside to show the imporant downward impact on all warming metrics that would result if it were shown to be a more reliable indication of aerosol forcing.

*c) Neglected anthropogenic forcing*

Left out from our analysis are other sources of anthropogenic forcing for which only the direct radiative forcing increase is reported in AR5: tropospheric ozone, stratospheric ozone, statospheric water vapour from $\,\,\mathrm{CH}_4$, surface albedo from land use changes, surface albedo from black carbon aerosol on snow and ice and contrails. They were neglected given the uncertainty on the shape of the response and their small combined impact. Their total should be positive and therefore, the median estimates of sensitivity presented in this paper will possibly be biased high by a small amount.

**Table 1:** Summary of the effective radiative forcing increase from 1750 to 2005 for different anthropogenic sources as reported in the IPCC AR5

Forcing sources considered	Lower 5%	Median	Upper 5%
$\hbox {CO}_{2}$	0.40	0.45	0.50
All GHG	0.64	0.71	0.78
Aerosol direct effects	$-$0.24	$-$0.13	$-$0.02
Aerosol indirect effects	$-$0.49	$-$0.19	$-$0.08
Total anthropogenic forcing	0.21	0.43	0.55

Forcing sources neglected	Lower 5%	Median	Upper 5%
Tropospheric ozone	0.06	0.11	0.16
Stratospheric ozone	$-$0.04	$-$0.01	0.02
Stratospheric water vapour from $NH_4$	0.01	0.02	0.03
Land use changes	$-$0.07	$-$0.04	$-$0.01
Surface albedo from black carbon	0.00	0.03	0.06
Contrails	0.002	0.003	0.11

Given are the median, the upper 5% bound and the lower 5% bound in units of $\varDelta F _{2 \times \,\,\mathrm{CO}_2}$

*d) Solar forcing*

The two main natural forcings are solar and volcanic, but others include natural aerosols such as mineral dust and sea salt which will not be considered as they are not externally forced and depend on the internal variability of the system.

**Fig. 4:**
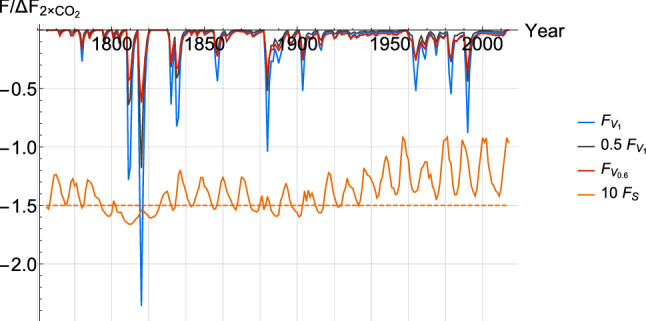
Volcanic forcing $F_{V_1}$ (blue) is shown alongside two damped versions. The black one is linearly damped by a constant 0.5 coefficient while the red one, $F_{V_{0.6}}$, is damped using Eq. 18 with $\nu =0.6$. The solar forcing $F_S$ (orange) has been shifted down by $-1.5$ and amplified by a factor of 10 for clarity

We use the recommended solar forcing $F_S$ for CMIP5 experiments shown in Fig. 4 (along with the volcanic forcing). It is reconstructed by regressing sunspot and faculae time series with total solar irradiance (TSI) (Wang et al. 2005). To obtain the solar perturbation to radiative forcing, the TSI is divided by 4 due to the spherical geometry of the Earth, multiplied by the average co-albedo of the Earth (about 0.7) and the average value of the two 11-year solar cycles from 1882 to 1904 is removed to obtain an anomaly. For the future, we take the solar cycle 23 (the last one before 2008) and reproduce it to extend the series as was recommended for the CMIP5 experiments.

*e) Volcanic forcing*

Contrary to other forcings, there was no standard volcanic forcing series prescribed for CMIP5 experiments. The volcanic forcing $F_V$ used here was derived from the optical depth $\tau _V$ using the approximate relation $F_V \approx -27 \,\,\mathrm{W\, m}^{-2} \tau _V$ for instantaneous forcing. The series for $\tau _V$ and the relation to forcing were obtained from the Goddard Institute for Space Science (GISS) website (Sato 2012). The volcanic forcing covers the period from 1850 to 2012 and it was kept null for its extension into the future as was prescribed for CMIP5 experiments. We extend it back to 1765 using the optical depth reconstruction of Crowley et al. (2008). To obtain the radiative forcing, the series was multiplied by a factor of $-24 \,\,\mathrm{W\, m}^{-2}$ so that the total forcing following the Pinatubo eruption from 1991 to 1996 is equal to the Sato (2012) dataset over the same period.

The response to volcanic forcing is crucial in improving the estimation of parameters, especially the scaling exponent *H*, as it provides insight into the climate system on fast timescales (inter-annual); within this scaling model, there is no characteristic scale and it has implications for longer timescales. Volcanic forcing is peculiar as it is strong and highly intermittent. The intermittency can be quantified by the parameter $C_1$ which corresponds to the fractal codimension (i.e. 1 minus the fractal dimension) characterizing the sparseness of volcanic “spikes” of mean amplitude (see Lovejoy and Schertzer 2013b; Lovejoy and Varotsos 2016), with large eruptions producing negative forcing spikes of magnitude greater than $\varDelta F _{2 \times \,\,\mathrm{CO}_2}$; on the other hand, the instantaneous temperature response is weaker than expected using linear response theory.

It was found that even though volcanic forcing dwindles away quickly, it has noticeable effects on the climate at decadal timescales and longer by sharply reducing the ocean heat intake (Church et al. 2005; Stenchikov et al. 2009). This, in fact, corresponds to the physical mechanism behind the long-range memory to forcing in the linear response framework, and it acts to reduce the instantaneous impact of the volcanic forcing. Gregory et al. (2016) found that this reduction in ocean heat intake explains most of the discrepancy between forcing and response, but also added that “the magnitude of the volcanic forcing [...] may be smaller in AOGCMs (by 30 % for the HadCM3 AOGCM) than in off-line calculations that do not account for rapid cloud adjustment”.

The volcanic response appears to be non-linear as the intermittency (“spikiness”, sparseness of the spikes) parameter $C_1$ changes from about ${C_1}_{F_V} \approx 0.2$ for the input volcanic forcing to ${C_1}_{T} \lesssim 0.1$ for the temperature response :the latter is therefore much less intermittent than the former. Theoretically, a linear response model with a power-law Green’s function cannot alter the intermittency parameter, although these estimates are sensitive to finite size effects and internal variability (Lovejoy and Varotsos 2016).

Since the volcanic forcing is too strong and too intermittent, using it within the SCRF framework requires the use of an effective volcanic forcing series if we are to use it in the linear response framework. The following theoretically motivated non-linear relation damps the amplitude of the volcanic forcing while also reducing its intermittency parameter :

18 $$\begin{aligned} \frac{F_{V_{\nu }}}{<F_V>}= \left[ \frac{F_V}{<F_V>} \right]^\nu  \end{aligned}$$

where $F_{V_{\nu }}$ is the damped effective volcanic forcing, $\nu$ is the damping exponent and $<F_V>$ is the mean $F_V$. The damping exponent required to reduce the intermittency parameter of the volcanic temperature response, ${C_1}_{T_V}$, can be theoretically calculated using the relation :

19 $$\begin{aligned} {C_1}_{F_V} \nu ^{\alpha _{MF}} ={C_1} _{T_V} \end{aligned}$$

where $\alpha _{MF}$ is the multifractality index of the volcanic forcing (e.g. Lovejoy and Schertzer 2013b). For $\alpha _{MF} \approx 1.6$, we find $\nu \lesssim 0.65$. This calculation might underestimate $\nu$, because the temperature variability also includes the response to the less intermittent forcing (anthropogenic and solar) as well as the internal variability, which is quasi-Gaussian with $C_1\approx 0$. Given the difficulty in estimating $C_1$ and $\alpha _{MF}$ to calculate $\nu$, we introduce $\nu$ as a free parameter and estimate it directly.

Another avenue would be to simply introduce a linear scaling factor to reduce the amplitude of the volcanic forcing, but this would not change the nondimensional spikiness. The two methods are approximately equal for medium size eruptions, but strong peaks get reduced further in the non-linear damping case (see Fig. 4). From the point of view of maximizing the variance explained by the forced response in the temperature record, the linear damping method would produce very similar result, but we choose the non-linear damping method simply because it is better justified theoretically and decreases the intermittency parameter $C_1$.

#### Surface air temperature data and cmip5 simulations

Our analysis was performed on 5 observational records of surface air temperature each spanning at least the period 1880–2014: HadCRUT4 (Morice et al. 2011), the Cowtan and Way reconstruction version 2.0 (C and W) (Cowtan and Way 2014), GISS Surface Temperature Analysis (GISTEMP) (Hansen et al. 2010; GISTEMP Team 2015), NOAA Merged Land Ocean Global Surface Temperature Analysis Dataset (NOAAGlobalTemp, formerly known as MLOST) (Smith et al. 2008) and Berkeley Earth Surface Temperature (BEST) (Rohde et al. 2013).

The HadCRUT4 dataset is a combination of the sea-surface temperature records compiled by the Hadley Centre of the UK Met Office with the land surface air temperature records compiled by the Climate Research Unit in East Anglia; the C and W dataset uses HadCRUTv4 as raw data, but aims to address coverage bias by infilling missing data by kriging; the dataset with land air temperature anomalies interpolated over sea-ice was used. GISTEMP is produced by the Goddard Institute for Space Studies by combining the Global Historical Climate Network version 3 (GHCNv3) land surface air temperature records with the Extended Reconstructed Sea Surface Temperature version 4 (ERSST) and the temperature dataset from the Scientific Community on Antarctic Research (SCAR). NOAA National Climatic Data Center also uses GHCNv3 and ERSST, but with different quality controls and bias adjustements. BEST uses its own land surface air temperature product combined with a modified version of HadSST.

The CMIP5 models used are presented in Table 2. The 32 selected GCMs have historical simulation outputs available for the period from 1860 to 2005 and outputs of scenario runs over 2005–2100 for RCP 2.6, RCP 4.5 and RCP 8.5.

**Table 2:** CMIP5 models used are presented here along with the modeling centres which produced them

Modeling Center (or Group)	Institute ID	Model Name
Beijing Climate Center, China Meteorological Administration	BCC	BCC-CSM1.1 $^{(1)}$
		BCC-CSM1.1(m) $^{(2)}$
College of Global Change and Earth System Science, Beijing Normal University	GCESS	BNU-ESM $^{(3)}$
Canadian Centre for Climate Modelling and Analysis	CCCMA	CanESM2 $^{(4)}$
National Center for Atmospheric Research	NCAR	CCSM4 $^{(5)}$
Community Earth System Model Contributors	NSF-DOE-NCAR	CESM1(CAM5) $^{(6)}$
Centre National de Recherches Météorologiques / Centre Europen de Recherche et Formation Avance en Calcul Scientifique	CNRM-CERFACS	CNRM-CM5 $^{(7)}$
Commonwealth Scientific and Industrial Research Organization in collaboration with Queensland Climate Change Centre of Excellence	CSIRO-QCCCE	CSIRO-Mk3.6.0 $^{(8)}$
EC-EARTH consortium	EC-EARTH	EC-EARTH $^{(9)}$
LASG, Institute of Atmospheric Physics, Chinese Academy of Sciences and CESS, Tsinghua University	LASG-CESS	FGOALS-g2 $^{(10)}$
The First Institute of Oceanography, SOA, China	FIO	FIO-ESM $^{(11)}$
NOAA Geophysical Fluid Dynamics Laboratory	NOAA GFDL	GFDL-CM3 $^{(12)}$
		GFDL-ESM2G $^{(13)}$
		GFDL-ESM2M $^{(14)}$
NASA Goddard Institute for Space Studies	NASA GISS	GISS-E2-H (p1$^{(15)}$, p2$^{(16)}$, p3$^{(17)}$)
		GISS-E2-R (p1$^{(18)}$, p2$^{(19)}$, p3$^{(20)}$)
National Institute of Meteorological Research/Korea Meteorological Administration	NIMR/KMA	HadGEM2-AO $^{(21)}$
Met Office Hadley Centre (additional HadGEM2-ES realizations contributed by Instituto Nacional de Pesquisas Espaciais)	MOHC (and INPE)	HadGEM2-ES $^{(22)}$
Institut Pierre-Simon Laplace	IPSL	IPSL-CM5A-LR $^{(23)}$
		IPSL-CM5A-MR $^{(24)}$
Japan Agency for Marine-Earth Science and Technology,	MIROC	MIROC-ESM $^{(25)}$
Atmosphere and Ocean Research Institute (The University of Tokyo), and National Institute for Environmental Studies		MIROC-ESM-CHEM $^{(26)}$
Atmosphere and Ocean Research Institute (The University of Tokyo), National Institute for Environmental Studies, and Japan Agency for Marine-Earth Science and Technology	MIROC	MIROC5 $^{(27)}$
Max-Planck-Institut für Meteorologie	MPI-M	MPI-ESM-MR $^{(28)}$
(Max Planck Institute for Meteorology)		MPI-ESM-LR $^{(29)}$
Meteorological Research Institute	MRI	MRI-CGCM3 $^{(30)}$
Norwegian Climate Centre	NCC	NorESM1-M $^{(31)}$
		NorESM1-ME $^{(32)}$

The superscript number in paranthesis identifies the GCMs for later references

### Parameter estimation

We have now presented the SCRF and we need to establish a procedure to estimate its parameters $s$, $\tau$, and *H* as well as the forcing parameters $\alpha$ and $\nu$. To achieve this, we relate the temperature and forcing data introduced above through the SCRF in a multi-parameter Bayesian estimation scheme.

The radiative forcing data is used to produce a theoretical forced temperature response by means of convolution with the SCRF. The convolution was implemented numerically as a discrete sum :

20 $$\begin{aligned} \varDelta T _{Forced}(t)= -s \frac{H}{\tau } \sum _{i=1}^N \left( \frac{t+\tau -{t'}_i}{\tau } \right) ^{H-1} F({t'}_i) \varDelta t' \end{aligned}$$

where $\varDelta T_F$ is the forced temperature response, *H* is the scaling exponent, $s$ is the sensitivity to integrated radiative forcing, $\tau$ is the high-frequency cutoff and $F(t_i)$ is the annual forcing series *F*(*t*) linearly interpolated to a resolution such that $\varDelta t'={t'}_{i+1}-{t'}_{i} =0.05 \, \,\,\mathrm{year}$; this resolution is much smaller than $\tau \approx 2 \, \,\,\mathrm{years}$ (see below) and it was found to be sufficient to produce a temperature response with negligible error compared to the analytic response for different polynomial forcing scenarios while remaining computationally efficient.

Due to the assumption of linearity, the forcing series used can be written as the sum of the constituent forcings :

21 $$\begin{aligned} F(t)=F_{GHG}(t)+\alpha F_{Aer}(t)+F_S (t)+F_{V_{\nu }} \end{aligned}$$

This includes the two extra parameters discussed earlier: the aerosol linear scaling factor $\alpha$ and the volcanic non-linear damping exponent $\nu$. This allows us to take into account the uncertainty on the forcing when estimating model parameters; the uncertainties on $F_{GHG}$ and $F_{S}$ are thus neglected, because they are overwhelmed by the uncertainty on $F_{Aer}$. The uncertainties add in quadrature and therefore, if we leave out the uncertainty on $F_{GHG}$when adding to the uncertainties of the forcing from the aerosol direct and indirect effects, we only underestimate the total uncertainty (at the 90% confidence level) by about $0.01\varDelta F_{2 \times \,\,\mathrm{CO}_2}$ (using the errors given in Table 1 for the total change over the 1750–2005 period). The amplitude of $F_V$ is sensitive to $\nu$ and its parameter spread is informative of the related uncertainty in the volcanic forcing.

All the series used for parameter estimation were adjusted to the same anomaly level so that their mean values were zero over the reference period of 1880–1910. There are thus five parameters to determine. A time-dependent forced response $\varDelta T_{Forced}(s ,H,\tau ,\alpha ,\nu ;t)$ is calculated for each parameter combination and removed from the temperature series to obtain a series of residuals which represent an estimator $\widehat{\varDelta T}_{Internal}$ of the historical internal variability.

22 $$\begin{aligned} \widehat{\varDelta T}_{Internal}(s ,H,\tau ,\alpha ,\nu ;t|\varDelta T_{Obs}) =\varDelta T_{Obs}(t)- \varDelta T_{Forced}(s ,H,\tau ,\alpha ,\nu ;t) \end{aligned}$$

where $\varDelta T_{Obs}$ is an observational temperature dataset. This allows us to calculate the likelihood function $\mathcal {L}$ to be maximized which corresponds to the probability *Pr* of the internal variability to follow our assumed error model :

23 $$\begin{aligned} \mathcal {L} (s ,H,\tau ,\alpha ,\nu |\varDelta T _{Obs})=Pr(\varDelta T _{Obs}|s ,H,\tau ,\alpha ,\nu ) \end{aligned}$$

The error model we use is a fractional Gaussian noise (fGn) with zero mean (see Lovejoy et al. 2015, 2017), and therefore the residuals are not independently distributed. This model approximates well the scale dependence of the internal variability, and so even if it is misspecified, it will be superior to non-parametric approaches (Poppick et al. 2017; Lovejoy et al. 2016).

Using Bayes’ rule, we can derive a probability distribution function (PDF) for the parameters of interest:

24 $$\begin{aligned} Pr(s ,H,\tau ,\alpha ,\nu |\varDelta T _{Obs})=\frac{Pr(\varDelta T _{Obs}|s ,H,\tau ,\alpha ,\nu ) \pi (s ,H,\tau ,\alpha ,\nu )}{Pr (\varDelta T _{Obs})} \end{aligned}$$

where $\pi (s ,H,\tau ,\alpha ,\nu )$ corresponds to the prior distribution for the parameters.

The prior distribution for the high-frequency cutoff $\tau$ is taken to be a Dirac-delta at 2 years. From the point of view of the parameter estimation, this choice has a marginal effect on the results since it is the ratio of *H* to $\tau$ which is most determinant for sensitivity estimates at equilibrium, and *H* is allowed to vary. This weak assumption makes the estimation procedure more manageable by reducing the number of free parameters to four : *H*, $s$, $\alpha$ and $\nu$. See Appendix B for a detailed discussion concerning this choice of $\tau$, and the sensitivity of the result to this choice.

Given no previous knowledge of $s$ and $\nu$, we simply assume a non-informative uniform prior over the range of parameters considered. We know that the scaling exponent *H* should be negative since the ECS would diverge if $H \ge 0$, and therefore, we need a prior in *H* which discards such non-physical values. For each value of *H*, there is a unique TCR to ECS ratio which can be calculated (shown on Fig. 2). We make use of this fact to derive a prior in *H* based on the TCR to ECS ratios from an ensemble of 23 CMIP5 GCMs (Yoshimori et al. 2016). In order to minimize the impact of this choice, we take a wide uniform distribution covering the mean ratio plus or minus 4 times the standard deviation, i.e. between 0.22 and 0.91, which yields a corresponding uniform distribution in H between $-1.1$ and $-0.1$. Regarding $\alpha$, we take as the prior distribution a normal distribution *N*(1.00, 0.55) which has a 90% CI coherent with the modern value for $F_{Aer}$ from the IPPC AR5, $[-1.9,-0.1] \,\,\mathrm{W\, m}^{-2}$, since the modern value of $F_{Aer} \approx -1.0 \,\,\mathrm{W\, m}^{-2}$ in the series we used. The efficacy of the parameter estimation method is tested on synthetic temperature series in Appendix C.

## Results

### Scaling climate response function

In this section, using Bayes’ theorem as described above, we derived a PDF for the parameters of the SCRF from the mean likelihood of the five observational datasets available: HadCRUTv4, C&W, GISTEMP, NOAAGlobalTemp and BEST. Two different series were used for the aerosol forcing in equation 21: $F_{Aer_{RCP}}$ and $F_{Aer_{Q_a}}$, and the results are compared. Estimates and CIs are rounded to the resolution used for the likelihood function of the variable.

**Fig. 5:**
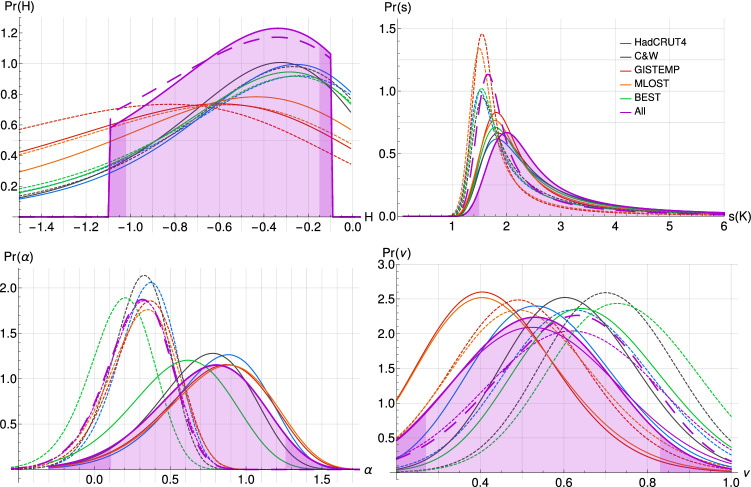
For each observational dataset and also their average, PDFs are shown for the scaling exponent *H* (top left), the equilibrium climate sensitivity *s* in *K* per doubling of CO_2_ (top right), the volcanic damping exponent $\nu$ (bottom right), and the aerosol linear scaling factor $\alpha$ (bottom left); for each case the probabilities over the remaining parameters were integrated out. Shown alongside are the corresponding PDFs for the parameter estimation based on both ${F_{Aer}}_{RCP}$ (solid) and ${F_{Aer}}_{Q_a}$ (dashed). The average PDFs (purple) from the five observational datasets are shown after the prior distribution has been applied; the one using ${F_{Aer}}_{RCP}$ is shown as the main result with shading and darker $5 \%$ tails

The PDF for the aerosol linear scaling factor $\alpha$, using $F_{Aer_{RCP}}$ (Fig. 5: bottom left, solid line), yields a 90% CI of [0.1, 1.3] with median at 0.8; for comparison, a uniform prior in $\alpha$, and a prior consistent with S15, would yield the median values of 0.6 and 0.7 with CIs of [0.2, 1.2], and [0.4, 0.9] respectively; the posterior result is therefore not sensitive to the prior assumption. Given that in 2005 $F_{Aer_{RCP}} \approx -1 \,\,\mathrm{W\, m}^{-2}$, the negative value of $\alpha$ translates into confidence intervals for the modern aerosol forcing. It is interesting to note that we recovered a 90% CI close to what had been argued by S15, namely $[-1.0,-0.3] \,\,\mathrm{W\, m}^{-2}$, thus reinforcing his claim to decrease the uncertainty on aerosol forcing using a completely independent approach, although more comprehensive estimates still support the wider range from the IPCC’s AR5 (Bellouin et al. 2020). On the other hand, the result using $F_{Aer_{Q_a}}$ supports a significantly weaker (better constrained) aerosol forcing with an $\alpha$ median value at 0.2 and a 90% of $[-0.2,0.6]$ (Fig. 5: bottom left, dashed). The sulfur dioxide emission series $Q_a$, and therefore ${F_{Aer}}_{Q_a}$, shows a sharp increase between 1950 and 1980, sharper than ${F_{Aer}}_{RCP}$. Meanwhile, the global mean temperature only decreases marginally and therefore, $\alpha$ for ${F_{Aer}}_{Q_a}$ needs to be smaller for a good agreement.

The volcanic damping exponent $\nu$ was found (when using $F_{Aer_{RCP}}$) to have a 90% CI of [0.25, 0.85] with median value at 0.55 (Fig. 5: bottom right, solid line) and using $F_{Aer_{Q_a}}$ yielded a slightly higher median $\nu$ of 0.60 with a 90% CI of [0.30, 0.90] (Fig. 5: bottom right, dashed line). The datasets which tend towards a lower $\nu$ (i.e. smoother volcanic forcing), namely NOAAGlobalTemp and GISTEMP, are also those with stronger statio-temporal filtering, and therefore, a smoother volcanic response. These results confirm that volcanic forcing is generally overpowered since $\nu =1$ has practically null probability in both cases. This means that using the original volcanic forcing series described above without the non-linear damping does not reproduce well, within the SCRF model presented, the cooling events observed in the instrumental records following eruptions: the cooling would be too strong. It also shows that the volcanic contribution is essential since $\nu$ values near zero, which effectively corresponds to a constant (weak) mean volcanic forcing, are also ruled out. We also obtain from Eq. 19, taking $\alpha _{MF} \approx 1.6$ and ${C_1}_{F_V} \approx 0.2$, that the intermittency parameter $C_1$ of the effective volcanic forcing, and also the linear volcanic temperature response, is ${C_1}_{T_V} = 0.07_{-0.05}^{+0.08}$ at the 90% confidence level for the $F_{Aer_{RCP}}$ result, and ${C_1}_{T_V} = 0.09_{-0.06}^{+0.07}$ for the $F_{Aer_{Q_a}}$ result.

The most crucial parameter in our model is its scaling exponent *H* which is the main determinant for ECS estimates made later. We found, when using $F_{Aer_{RCP}}$, a 90% CI of $[-1.0,-0.1]$, with median value at $-0.5$ (Fig. 5: top left, solid line). Using $F_{Aer_{Q_a}}$ did not significantly change the median estimate of *H* and the 90% CI (Fig. 5: top left, dashed line). The purpose of *H* is somewhat similar to that of an ocean diffusivity parameter in a one-dimensional SCM and we see that in fact, the datasets using HadSST: HadCRUTv4, C&W and BEST, yielded PDFs closer to each others than to those using ERSST: NOAAGlobalTemp and GISTEMP.

**Fig. 6:**
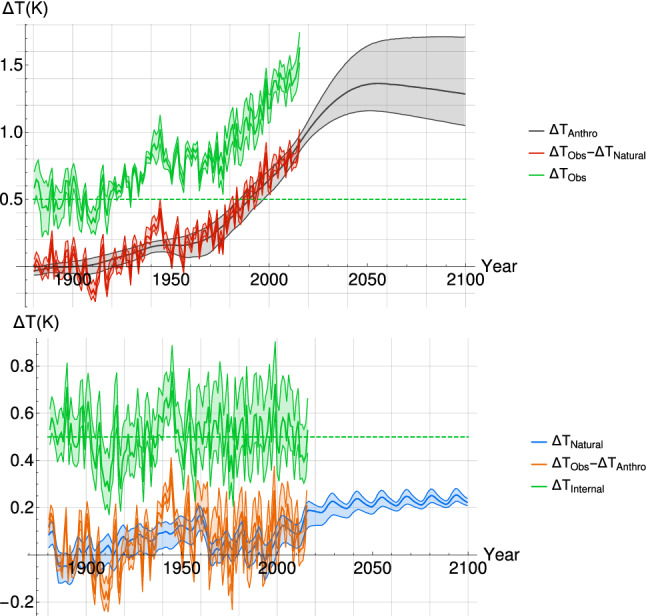
(top) The mean observational temperature series $\varDelta T_{obs}$ (green and shifted up by 0.5 K) has $\varDelta T_{Natural}$ removed and the residual $\varDelta T_{Obs}-\varDelta T_{Natural}$ (red) is compared with $\varDelta T _{Anthro}$ (black) calculated using $F_{Aer_{RCP}}$; they are highly correlated (r=0.94). (bottom) $\varDelta T_{Anthro}$ is removed from $\varDelta T_{Obs}$ and the residual $\varDelta T_{Obs} -\varDelta T _{Anthro}$ (orange) is compared with $\varDelta T_{Natural}$ (blue); the are correlated with r = 0.51. Once $\varDelta T_{Natural}$ is also removed, we obtain $\varDelta T_{Internal}$ (green and shifted up by 0.5 K), which is thus uncorrelated with $\varDelta T_{Natural}$. The error (shaded) given for each curve correponds to the 90% CI derived from the estimated parameters, except in the case of the observational data where it correponds to the spread between the datasets (1.645 standard deviation, by analogy with a 90% CI)

We can therefore identify and separate the anthropogenic and natural components of the forced variability, $\varDelta T_{Anthro}$ and $\varDelta T_{natural}$ respectively, from the observational temperature series $\varDelta T_{Obs}$ to obtain the internal variability $\varDelta T _{Internal}$, see Fig. 6.

25 $$\begin{aligned} \varDelta T _{Internal}(t) = \varDelta T_{Obs}(t)-\varDelta T_{Anthro}(\lambda ,H,\tau ,\alpha ,t)-\varDelta T _{Natural}(H,\lambda ,\tau ,\nu ,t) \end{aligned}$$

$\varDelta T_{Anthro}$ and $\varDelta T _{Natural}$ are obtained by the convolution of the associated forcing with the SCRF; solar and volcanic forcing in the case of $\varDelta T _{Natural}$, and greenhouse gases and aerosol forcing in the case of $\varDelta T_{Anthro}$. Our result confirms that much of the 20$^{\mathrm{th}}$ century warming is indeed human-induced, while much of the decadal scale variability can be attributed to natural forcing, and internal processes. In the projection period, after 2015, $\varDelta T_{Natural}$ brings a positive contribution to the temperature anomaly since the prescribed solar forcing is a repetition of the anomalously high solar cycle 23. In reality, the natural forcing for the future will be quite different than those prescribed here for conformity with CMIP5 experiments.

### Climate sensitivity

The paleoclimate record has been used to estimate a likely range for ECS (equivalent to our parameter *s*) by comparing the temperature and forcing changes over past periods such as the last millennium (Hegerl et al. 2006: [1.9, 4.3] K) and the last glacial maximum (LGM) (Schmittner et al. 2011:[1.4, 3.5] K; von der Heydt et al. 2014: [2.0, 2.6] K) There is evidence that the climate sensitivity depends on the mean climate state and therefore the modern ECS does not necessarily correspond to past ECS (Crucifix 2006; Yoshimori et al. 2011). In fact, there are many methodological challenges in order to fairly compare those different estimates, and in their extensive review on the subject, PALEOSENS Project Members (2012) considered 21 different paleoclimate studies in order to derive a [2.2, 4.8] K likely range for ECS.

An alternative approach developed to obtain ECS from the instrumental period are one-dimensional simple climate models (SCMs) with few parameters, among which the ECS is specified rather than being measured as an emergent property. The outputs of SCMs are then evaluated against the observational record, usually within a Bayesian framework, to find the best parameter combination and derive a range for the ECS : [2.1, 7.1] K (Meinshausen et al. 2009), [1.2, 3.5] K (Aldrin et al. 2012), [1.5, 5.2] K (Bodman et al. 2013), [0.9, 3.2] K (Skeie et al. 2014) and [1.9, 3.3] K (Johansson et al. 2015). See Bodman and Jones (2016) for a thorough review of this approach.

Recently, Sherwood et al. (2020) have conducted a comprehensive studies of all those lines of evidence and concluded that an ECS below 2 K was hard to reconcile with feedback-, historial- and paleoclimate-based estimates, while estimates paleoclimate-based estimates provide the strongest evidence against an ECS above 4.5 K. Their Bayesian analysis yielded a likely range for ECS of [2.6, 3.8] K, with 3.1 K at median, and a very like range [2.3, 4.7] K.

Similarly to SCMs, with probabilistic estimates of our model parameters it is straightforward to calculate the associated temperature response to any forcing scenario; this allows us to derive PDFs for common measures of climate sensitvity: TCR and ECS. In addition, we define $\hbox {ECS}_{500}$ as the temperature change 500 years after a step-function doubling in carbon dioxide concentration instead of at infinity.

**Fig. 7:**
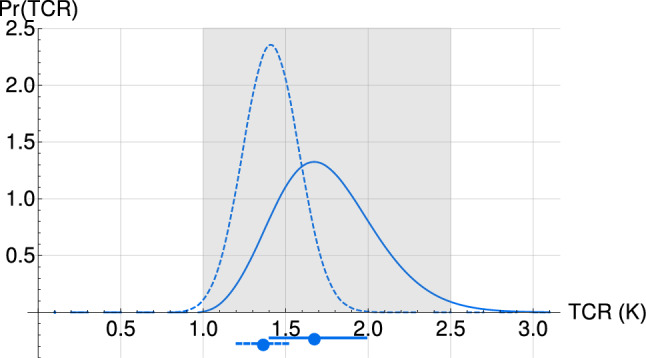
The PDF for TCR is calculated using $F_{Aer_{RCP}}$ (solid) and $F_{Aer_{Q_a}}$ (dashed). The associated likely intervals (66%) (bars under the axis) are tighter than the IPCC likely range (gray shading) with lower median

Using Eq. A9 for each parameter combination with the associated probability assigned, we derived the PDFs for TCR shown in Fig. 7 using a uniform prior in TCR. Our result is slightly lower and better constrained than the one given in the IPCC AR5: a $[1.0,2.5] \, \,\,\mathrm{K}$ likely range with best value at around 1.8 K. Using $F_{Aer_{RCP}}$, we found a median TCR of 1.7 K with a likely range of [1.4, 2.0] K, and when using $F_{Aer_{Q_a}}$ the median is revised downward to 1.3 K with an even slimmer likely range of [1.1,1.5] K.

**Fig. 8:**
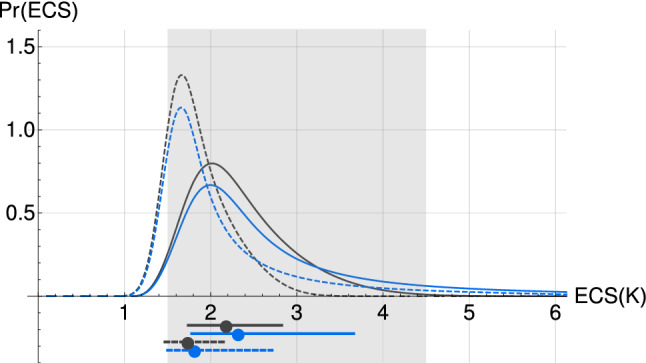
Same as Fig. 7, but for ECS (blue) and $\hbox {ECS}_{500}$ (black)

The PDFs for ECS (Fig. 8) are calculated with a uniform prior distribution in ECS and a likely range subset of the corresponding IPCC AR5 likely range of [1.5,4.5] K was found. Using ${F_{Aer}}_{RCP}$, the ECS PDF found is skewed towards higher ECS with a likely range of [1.8,3.7] K with its median at 2.3 K. The result for ${F_{Aer}}_{Q_a}$ yields a likely range of [1.5,2.7] K with its median at 1.8 K. Both results are coherent with the IPCC AR5 likely range, albeit on its lower side. A large fraction of the expected warming for the models with very high ECS only occurs at long timescale as a consequence of the slow convergence time when $H \rightarrow 0^-$. This is obvious when considering $\hbox {ECS}_{500}$, calculated with Eq. A5, for we obtain more symmetrical distributions than for ECS: a likely range of [1.7, 2.8] K with its median at 2.2 K for ${F_{Aer}}_{RCP}$, and a likely range of [1.5, 2.1] K with its median at 1.7 K. The median ECS we found when using $F_{Aer_{RCP}}$, which is derived from GCM experiments, is 0.6 K higher than when using $F_{Aer_{Q_a}}$ which is entirely based on historical observations. The latter is close to other observation-based estimates with low-ECS (Ring et al. 2012; Skeie et al. 2014) while the former is closer to high-ECS observation-based estimates (Meinshausen et al. 2009; Bodman et al. 2013; Otto et al. 2013; Johansson et al. 2015) as well as that of Sherwood et al. (2020), but both are lower than the 3 K best value reported in AR5 which is very close to CMIP3 and CMIP5 GCMs based estimates. All the ECS results are summarized in Table 3

**Table 3:** Summary of ECS from other observational studies

Source	Mean	Median	Likely (17–83%)
IPCC			
First Assessment Report (1990)		2.5	1.5–4.5
Second Assessment Report (1995)		2.5	1.5–4.5
Third Assessment Report (2001)			1.5–4.5
Fourth Assessment Report (2007)		3	2–4.5
Fifth Assessment Report (2013)			1.5–4.5
Feedback framework			
Gregory et al. (2002)		6.1	1.6-$\infty$
Lindzen and Choi (2011)	0.7		0.6–1.0
Otto et al. (2013)	2.0		1.2–3.9
SCMs			
Meinshausen et al. (2009)			2.1–7.1
Ring et al. (2012)	1.5–2.		
Bodman et al. (2013)	3.2	3.1	1.5–5.2
Skeie et al. (2014)	1.8	1.7	0.9–3.2
Johansson et al. (2015)	2.6		1.9–3.3
Comprehensive			
Sherwood et al. (2020)		3.1	2.6–3.8
Current study when using $F_{Aer_{RCP}}$			
$\hbox {ECS}_{500}$	2.3	2.2	1.7–2.8
ECS(Uniform Prior)	2.8	2.3	1.8–3.7
Current study when using $F_{Aer_{Q_a}}$			
$\hbox {ECS}_{500}$	1.9	1.7	1.5–2.2
ECS(Uniform Prior)	2.2	1.8	1.5–2.7
GCMs			Very likely (5–95%)
CMIP3 (AR4 Table 8.2)	3.2	3.2	2.1–4.4
CMIP5 (AR5 Table 9.5)	3.22	2.89	1.9–4.5

Some results were taken directly from the summaries in Bodman et al. (2016) and Lewis and Crok (2014)

### Projections to 2100

Using Eq. 20, we are now able to use the SCRF to reconstruct the forced temperature variability over the historical period and make projections for the coming century according to the RCP scenarios. We compare these purely observations-based projections with those from the CMIP5 MME considered (32 GCMs). The CI given for the MME correspond to the spread between the different GCMs, i.e., the structural uncertainty, since a full probabilistic characterization of uncertainty is generally not possible with GCMs given the hundreds of degrees of freedom involved. In addition, we also show the SCRF projections after recalibrating the sensitivity coefficienct $s$ on the historical part of the MME, thus confirming that its forced response is also linear and that the memory in the MME projections is close to that of the SCRF. Furthermore, the ability of the SCRF method was shown for the majority of individual CMIP5 models by calibrating parameters over the low-emission scenario RCP 2.6 and projecting the medium- and high-emission scenarios, RCP 4.5 and RCP 8.5 (Hébert 2017).

**Fig. 9:**
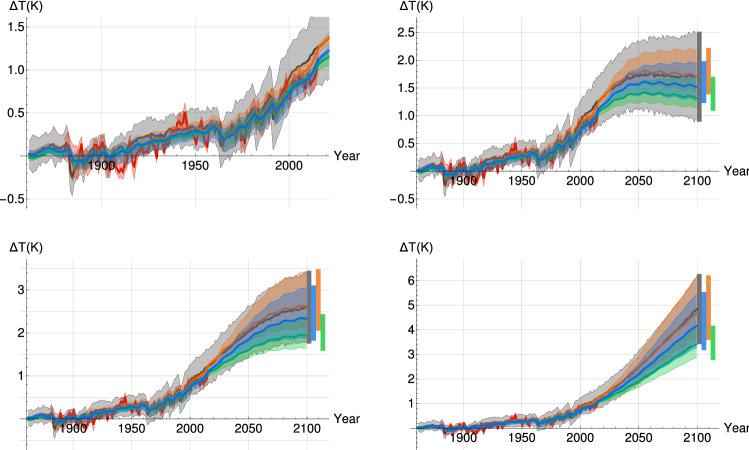
The median forced temperature variability is projected using the SCRF, with the parameters calculated using $F_{Aer_{RCP}}$ (blue) or $F_{Aer_{Q_a}}$ (green), and compared with the CMIP5 MME projection (black) and a SCRF projection of the MME (orange) as well as the historical temperature observations (red); 90% CI are indicated along the projections (shaded) and for the year 2100 (thick vertical lines). The historical period (top left) and the projections until 2100, for RCP 2.6 (top right), RCP 4.5 (bottom left) and RCP 8.5 (bottom right), are shown

Over the 1860–2000 period, the reconstructed forced temperature variability produced by the SCRF, whether using ${F_{Aer}}_{RCP}$ or ${F_{Aer}}_{Q_a}$, and the mean of the CMIP5 MME track each other closely (Fig. 9, top left). There is only a small gap between the two over the 1915–1960 period when the CMIP5 MME is consistently warmer, but generally by less than 0.05 K. The variations of the instrumental data around the mean forced components, i.e. the internal variability, are greater in the first half of the 20th century than in the second. Between 1905 and 1935, the internal variability shows a prolonged negative fluctuation under the mean forced variability, and then a large positive fluctuation until 1945. Subsequently and until 2015, the departures from the mean are less persistent.

After 2000, the SCRF reconstruction accurately follows the so-called hiatus while the CMIP5 MME overshoots. This was also shown by Lovejoy (2015) with a simple Dirac CRF and an effective sensitivity to $\hbox {CO}_{2}$. In Schmidt et al. (2014), the overshoot of the CMIP5 MME is attributed to a combination of conspiring factors, mainly errors in volcanic and solar input, in representation of aerosols and in the evolution of El-Niño. In fact, an impulse-response model, similar to what we are using here, is used by Schmidt et al. (2014) to accurately retro-adjust the CMIP5 projection ensemble. We did not investigate the effect of those adjustments on the CMIP5 MME for future projections and simply considered the original GCM results.

The low-emission scenario, RCP 2.6, is of particular interest since the dominant anthropogenic forcing starts decreasing around the mid-2040s and allows us to observe the “warming in the pipeline” (Hansen et al. 2011) due to the large thermal inertia of oceans, which is modeled here by the long-range memory to radiative forcing of the SCRF. This means that the upper bound of the projection (higher H) keeps increasing up to 2100 even though the forcing has been decreasing since 2045, while the lower bound (lower H) has less memory and begins to decrease as early as 2055. The median SCRF surface temperature projection for $F_{Aer_{RCP}}$ (or $F_{Aer_{Q_a}}$; hereafter, the SCRF result using ${F_{Aer}}_{Q_a}$ is given in brackets after the result using ${F_{Aer}}_{RCP}$) under RCP 2.6 exhibits a behaviour close to a stabilization over the 2043–2100 period, reaching, in 2100, $1.5^{+0.4}_{-0.2}$ K (or $1.3 ^{+0.3} _{-0.2} K$) at the 90% confidence level compared to $1.7 \pm 0.8 K$ for the MME (Fig. 9, top right).

The behaviour of the SCRF projection is in fact similar to the MME result over 2043–2100 in that regard, which has an average projection which also shows stabilization, and thus we can project the MME rather well after recalibrating the sensitivity coefficient $s$. Out of the 32 CMIP5 models analyzed and numbered for reference in Table 2, 12 showed a significant cooling trend between 2043 and 2100 (2, 5, 10, 11, 13, 15, 16, 18, 22, 24, 28, 29) while 9 showed a significant warming trend over the same period (4, 6, 7, 8, 12, 21, 25, 26, 30); the remaining 11 models did not show a significant trend in either direction.

The forcing of the middle scenario, RCP 4.5, stabilizes in the mid 2060s, but the temperature projections, in Fig. 9 (bottom left), continue rising until 2100, both with the SCRF and the CMIP5 MME, and reaching $2.3 ^{+0.7}_{-0.5}K$ (or $1.9_{-0.3}^{+0.4}K$), at the 90 % confidence level, and $2.6 \pm 0.8 K$ respectively. Both projections for the business as usual scenario, RCP 8.5, show warming at a staggering rate up to $4.1 ^{+1.3}_{-0.9}K$ (or $3.4 ^{+0.7}_{-0.5} K$) and $4.8 \pm 1.3 K$, respectively, in 2100 as shown on Fig. 9 (bottom right). For the RCP 4.5 and RCP 8.5 scenarios, the SCRF projection has 25% (44%) and 15% (46%) less uncertainty than the MME spread and the median is colder by 0.3 K (or 0.7 K) and 0.7 K (or 1.4 K) respectively. In fact, the SCRF projection approximately corresponds to the lower half (or the lower quarter) of the CMIP5 MME projection range.

**Fig. 10:**
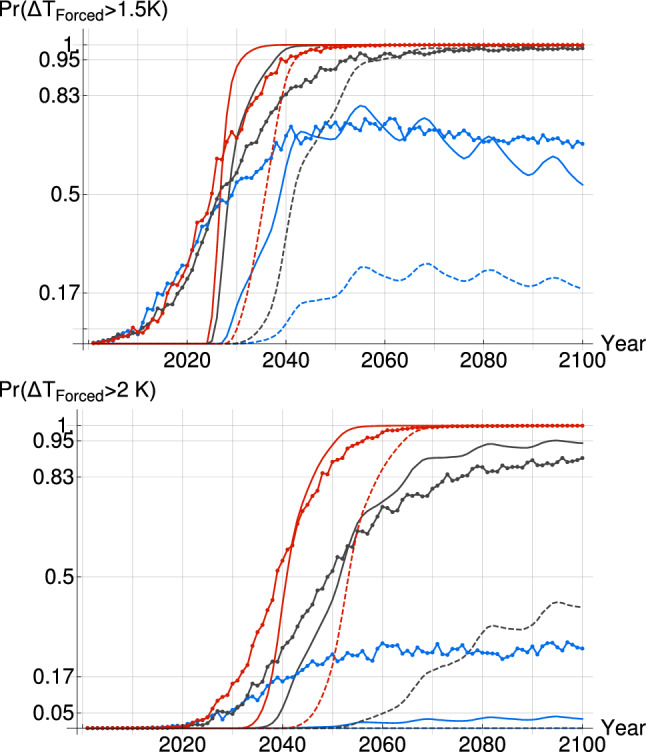
The probability for the global mean surface temperature of exceeding a 1.5 K threshold (top), and a 2 K threshold (bottom) are given as a function of years for the SCRF, using ${F_{Aer}}_{RCP}$ (solid) and using ${F_{Aer}}_{Q_a}$ (dashed), and for the CMIP5 MME (circles). The three RCP scenarios are considered for each case: RCP 2.6 (blue), RCP 4.5 (black) and RCP 8.5 (red)

International negotiations often invoke 1.5 K and 2 K threshold not to be crossed in order to avoid a major climatic upheaval. The probability when those temperature are reached under each scenario can be calculated for the SCRF and the CMIP5 MME (Fig. 10). For the latter, we assumed the global mean temperature of the MME every year to be normally distributed in order to obtain the probability of having crossed the given threshold at that time. Generally, the increase in probability as a function of years is sharper for the SCRF than for the CMIP5 MME given the smaller uncertainty on the projections.

For the low-emission scenario RCP 2.6, it is likely that the 1.5 K threshold would be exceeded in 2100 according to the SCRF projections with 54% probability (or 19%), while it was slightly more likely for the CMIP5 MME with 67% probability. It is very likely (> 97%) that the 2 K threshold would not be crossed in 2100 according to the SCRF projection while the CMIP5 MME still shows 26% probability of exceeding.

For the medium-emission scenario RCP 4.5, the SCRF asserts it is extremely likely (> 95%) that the global temperature will be above 1.5 K as early as 2038 (or 2059); for the CMIP5 MME, it becomes extremely likely after 2050. The 2 K threshold on the other hand will not as certainly be crossed in 2100 according to the SCRF projections as it has 94% (or 40%) of overshooting, similarly to the CMIP5 MME which shows a 89% probability of overshoot in 2100.

For the high-emission scenario RCP 8.5, all methods show a high probability of a warming exceeding 2 K before 2100. According to the SCRF, the risk of overshooting 1.5 K is negligible before 2024 (or 2028), but extremely likely after 2036 (or 2047), similarly to the CMIP5 MME which reaches the 95% probability of overshooting 1.5 K in 2038. The 2 K threshold is also extremely likely to be crossed about 15 years later in 2055 for both the SCRF and CMIP5 MME (or 2068).

## Conclusion

Multidecadal climate projections rely almost exclusively on deterministic global climate models (GCMs) in spite of the fact that there are still very large structural uncertainties between Coupled Model Intercomparison Project phase 5 (CMIP5) GCMs, i.e. each has its own climate, rather than the real world climate. Climate skeptics have argued that IPCC projections are untrustworthy precisely because they are entirely GCM based. While this conclusion is unwarranted, it underscores the need for independent and qualitatively different approaches. It is therefore significant that the alternative GCM-free approach we present here yields comparable results albeit with smaller uncertainty.

This motivated us to elaborate a model, based on the scaling of climate processes, for the response of the global mean air surface temperature of the Earth to external forcing: the scaling climate response function (SCRF). The forced component of temperature variability is reconstructed from external forcing within the linear response framework with a power-law scaling Green’s function truncated at high-frequency. The stochastic component ultimately due to the internal turbulent dynamics of the atmospheric system is approximated by a fractional Gaussian noise process, as was proposed in the ScaLing Macroweather Model (SLIMM) (Lovejoy et al. 2015). Similarly, GCMs yield stochastic internal variability with an approximately linear mean forced response (Meehl et al. 2004), and we showed that in fact the SCRF model can project their forced response rather accurately.

Our model is robust and by restricting the parameter space, it allows for a full probabilistic characterization of uncertainty by Bayesian inference. A by-product of our analysis is a better constrained aerosol forcing since we found the aerosol linear scaling factor $\alpha$ to be within a 90 % CI of [0.1, 1.3] for the RCP aerosol forcing $F_{Aer_{RCP}}$. This supports a revision of the global modern aerosol forcing 90 % confidence interval to a narrower $[-1.3,-0.1] \,\,\mathrm{W\, m}^{-2}$, similar to Stevens (2015). On the other hand, we obtain a very weak aerosol forcing if instead we use $F_{Aer_{Q_a}}$, which was reconstructed directly from sulfur dioxide emissions using a linearized version of Stevens’ proposed model (Eq. 16). While the difference between the aerosol series might arise from a misreprensation of aerosol effects in GCMs which were used to produce ${F_{Aer}}_{RCP}$, errors could also arise because of deviations from linearity with respect to $SO_2$ emissions due to other aerosol species not explicitly taken into account to produce ${F_{Aer}}_{Q_a}$.

Following others (Church et al. 2005; Stenchikov et al. 2009; Lovejoy and Varotsos 2016), we also found that the volcanic forcing was generally over-powered and overly intermittent, or too “spikey”, to produce results, within the SCRF framework, consistent with instrumental data. An effective volcanic forcing with lower-intermittency was obtained with a non-linear damping by the exponent $\nu$. It was found to be within [0.25,0.85] at the 90% confidence level using ${F_{Aer}}_{RCP}$ (or [0.30,0.90] using ${F_{Aer}}_{Q_a}$), yielding a corresponding CI of [0.02,0.15] (or [0.03,0.16] using ${F_{Aer}}_{Q_a}$) for the intermittency parameter $C_1$ of the effective volcanic forcing, which is compatible with the lower intermittency of the temperature.

Our analysis supports better constrained TCR and ECS likely range than the IPCC AR5. When using $F_{Aer_{RCP}}$ (or $F_{Aer_{Q_a}}$), the range shrinks from [1.0, 2.5] K to [1.4, 2.0] K for the TCR (or [1.2, 1.5] K) and from $[1.5,4.5] \, K$ to $[1.8,3.7] \, K$ for the ECS (or [1.5, 2.7] K); the median estimates also decrease from 1.8 K to 1.7 K (or 1.4 K) for the TCR and from 3.0 K to 2.4 K (or 1.8 K) for the ECS. This agrees with other recent observation-based studies (Otto et al. 2013; Skeie et al. 2014, and Johansson et al. 2015) which also support a downward revision of the ECS upper 17% bound by at least half a degree. In addition, the ECS$_{500}$ was found to be significantly smaller, $2.2_{-0.5}^{+0.6}K$ (or $1.7_{-0.2}^{+0.4}K$), than the ECS. This implies that if the ECS is on the higher end of the CI, then a large fraction of the warming would be experienced hundreds of years after a potential stabilization of anthropogenic forcing. An important and rather conservative claim supported by this evidence is therefore that the upper 5% ECS bound and median of AR5 can be safely revised downward to 4.0 K and 2.5 K. The lower 5% bound of 1.5 K, on the other hand, remains reliable.

Our ECS likely range is therefore better constrained than paleoclimate estimates such as Hegerl et al. (2006) and PALEOSENS Project Members (2012) who found $[1.9,4.3]\, K$ and $[2.2,4.8]\, K$ respectively. Our method is strictly based on modern instrumental data and the low uncertainty could be an “epoch bias”, i.e. if climate sensitivity depends on the climate mean state, then an estimate based on only one period would not necessarily be representative.

Our historical approach also decreases the uncertainty on projections by more than a factor of two, even with the large uncertainty associated with aerosol forcing, compared to the structural uncertainty of a multi-model ensemble (MME) of CMIP5 global climate models for RCP scenarios. However, the uncertainties compared are qualitatively different since it is not possible to charaterize probabilistically the entire set of parameters involved in GCMs. The structural uncertainty in a MME is rather based on the dispersion between the GCM runs, each produced with a definite set of parameters. Our approach on the other hand does not yield any structural uncertainty since we assumed a single CRF which is able to produce a wide range of climate responses.

The SCRF projections to 2100 are entirely independent of the GCMs. Still, they are within the uncertainty bounds of the latter, effectively providing an independent confirmation of the GCM projections. This eliminates one of the key climate skeptic arguments: projections are not reliable since they are solely GCM-based. This conclusion is therefore important not only for scientists but also for policy makers, stressing the need for mitigation and adaptation measures. The SCRF is a the first of a new family of approaches that directly model the temperature at monthly scales and longer, see for example Procyk et al. (2020) that, in addition to scaling, directly exploits the principles of energy-balance (the fractional energy-balance equation) and produces projections and climate sensitivity estimates in a similar range, although with a better estimation of the scaling exponent *H* by also utilizing the macroweather high-frequency response.

According to our projections made to 2100, to avert a 1.5 K warming, future emissions will be required to undergo drastic cuts similar to RCP 2.6, for which we found a 46% probability to remain under the said limit; it is virtually certain that RCP 4.5 and RCP 8.5-like futures would overshoot. Even a 2.0 K warming limit would surely be surpassed by 2100 under RCP 8.5 and probably also under RCP 4.5, with only a 6% chance of remaining under the limit. The safest option remains RCP 2.6 which we project to remain under 2.0 K with very high confidence. The question remains whether it is at all realistic given that it relies strongly on the massive deployment of speculative negative emission technologies.

On the other hand, our model has obvious limitations since it assumes a linear stationary relationship between forcing and temperature, neglecting nonlinear interactions which could arise as the system evolves, as it currently warms. In particular, so-called tipping points could be reached in the coming century which would lead to a breakdown of the linear model proposed. Such potential behaviours are of critical value for improving future projections, but they have not yet been observed with high confidence even in GCMs. This underlines the need to exploit paleoclimate archives to achieve a better understanding of low-frequency natural variability, namely the transition scale from the macroweather regime to the climate regime. In this study, we have assumed the increased variability in the climate regime to be strictly a result of forcing, but internal modes of variability could also have a significant contribution for longer timescales.

More relevant to human activities and adaptation policies are regional projections which are also almost entirely produced by GCMs. In addition to the discrepancy between their global mean response, CMIP5 GCMs show widely varying spatial patterns of warming over the last century between themselves, and with significant differences from those observed in gridded instrumental temperature datasets (Hébert and Lovejoy 2018). Future work should explore the possibility of data driven models at the regional scale for climate projection. Already, it was found by Lovejoy and de Lima (2015) that in the macroweather regime, statistical space-time factorization holds for temperature and precipitation, both in instrumental datasets and in GCMs. This implies the possibility of developing linear response models for regional projections, although the main obstacle foreseen will be to identify the forced signal in the stronger regional internal variability.

## Figures and Tables

**Fig. 11:**
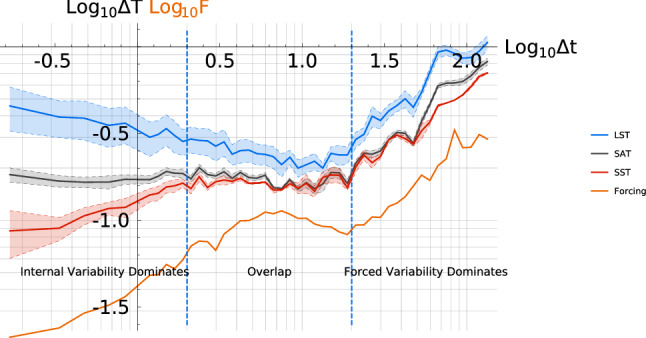
The mean Haar fluctuations (in units of K) are shown as a function of timescale $$\varDelta t$$ (in years), adjusted to annual resolution from monthly resolved series, for LST (blue), SAT (black) and SST (red), and the shaded areas correspond to the standard deviation between all the datasets considered. Vertical dashed lines indicate the domains over which internal or forced variability dominates, or where they are of similar magnitude. At high-frequency, the scaling behaviours differ due to diverging turbulent dynamics of the internal variability. The same analysis performed for the forcing series (orange; in number of $$CO_2 $$  doublings) shows a similar scaling behaviour at low-frequency, where the forced variability dominates, between the forcing and the temperature series. The series used are described in the data sub-section

**Fig. 12:**
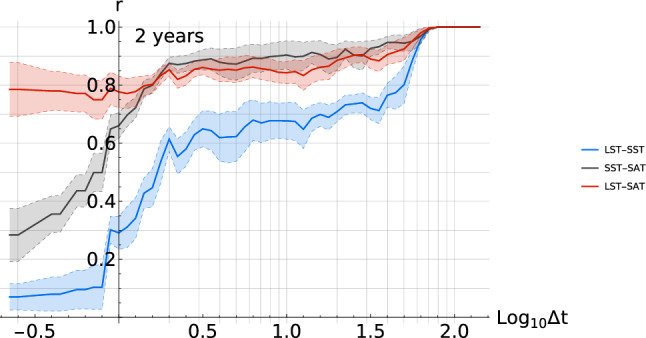
The average correlation coefficient *r* between the first-order Haar fluctuations of LST-SAT (red), SST-SAT (black), and LST-SST (blue) are shown as a function of timescale $\varDelta t$ in *years*. It was calculated for an ensemble of all possible pairs between the LST, SST and SAT datasets considered, and the shaded areas correspond to the one standard deviation. There is a rapid increasing trend in correlation between land and ocean as a function of timescale under 2 years and a slowly increasing trend above 2 years. To improve the statistics of the fluctuations at large timescale, the fluctuations from reversed series are also considered; this means there are only 2 fluctuations for timescales above half of the series length and only 1 or -1 correlation values are possible; thus the sharp increase above 40 years is not quantitatively significant

**Fig. 13:**
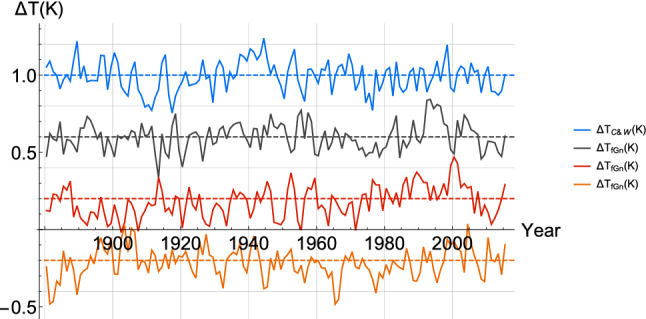
The forced temperature response is removed from the C&W instrumental temperature series to obtain the associated internal variability series (blue). The best volatility $\sigma$ and Hurst exponent $H'$ parameters to describe the series as an fGn process were estimated as 0.10 and $-0.26$ respectively. Three single realizations of an fGn process with the same parameters are shown (black, red and orange) are shown as examples. Each series has null mean and fluctuates about its associated reference line (dashed)

**Fig. 14:**
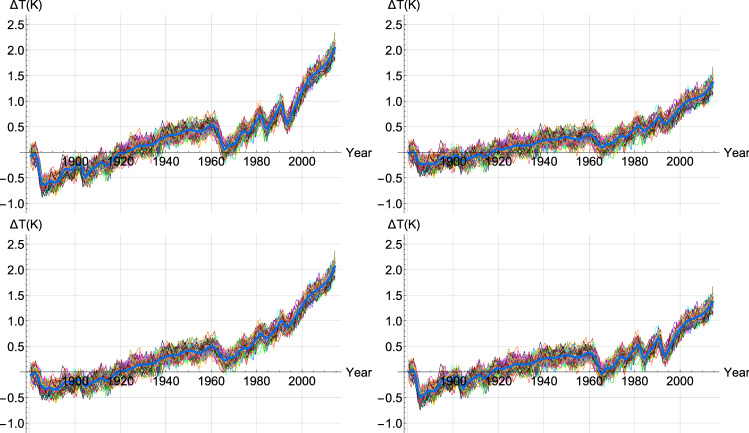
Each graph shows the simulated forced temperature response (thick, blue) with the following $(H,\nu )$ parameter combination : $(-0.2,0.8)$ (top left), $(-0.6,0.5)$ (top right), $(-0.2,0.5)$ (bottom left)and $(-0.6,0.8)$(bottom right). To each forced response, 25 realizations of a fGn were added (thin, colors) to produce synthetic series of known parameters

**Fig. 15:**
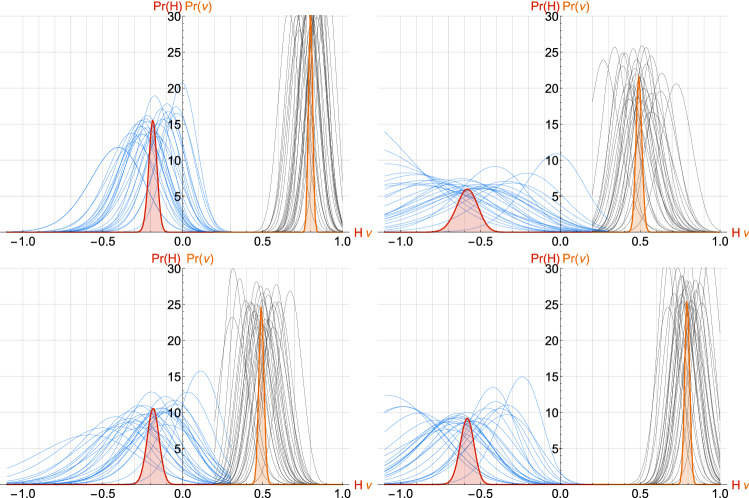
The resulting PDFs for each of the 25 realizations of fGn considered for the parameters *H* (blue) and $\nu$ (black) are shown for simulations produced with the following $(H,\nu )$ parameter combination : $(-0.2,0.8)$ (top left), $(-0.6,0.5)$ (top right), $(-0.2,0.5)$ (bottom left)and $(-0.6,0.8)$(bottom right). In addition, the re-normalized product of the individual PDFs, after they were re-interpolated at 0.01 increments, are shown for *H* (red) and $\nu$ (orange). The PDFs for the individual realizations have been multiplied by a factor of 5 for clarity

## Appendix: Response of the SCRF to theoretical forcing scenarios

In this section, we calculate the expected temperature response for different forcing scenario under the SCRF of Eq. 13. The expected forced temperature response for an $N^{th}$-order polynomial forcing function $F_{poly}=\sum \limits ^N _{i=0} a_i t^i \varTheta (t)$ can be written as:

A1 $$\begin{aligned} \varDelta T _{poly} (t)= & {} \int _{-\infty } ^t \sum \limits ^N _{i=0} a_i {t'}^i s \frac{(-H)}{\tau } \left( \frac{t-t'+\tau }{\tau } \right) ^{H-1} \varTheta (t') dt' \end{aligned}$$

A2 $$\begin{aligned} \varDelta T _{poly} (t)= & {} -s \frac{H}{\tau } \sum \limits _{i=0} ^N a_i \sum \limits _{k=0} ^i \left( {\begin{array}{c}i\\ k\end{array}}\right) \frac{(t+\tau )^{i-k}}{H+k} \frac{(-1)^k}{{\tau }^{H-1}}((t+\tau )^{H+k}-{\tau }^{H+k}) \end{aligned}$$

where $a_i$ is the $i^{\,\,\mathrm{th}}$-order coefficient of the forcing polynomial and the Heaviside function $\varTheta (t)$ makes the forcing null for negative times (the forcing is assumed to be zero for $t<0$). This is convenient not only since the ECS and TCR experiments are specific cases, but also because it also allows to calculate the theoretical response for any forcing which can be approximated by a polynomial. Of course, any orthogonal basis of functions could be used. Note that $\forall H \in \{0, -1, -2,..., -N \}$, where *N* is the order of the polynomial, there is a singularity, but the limit at such points is the same from both sides so that we can redefine it the value of the limit without further complications.

To measure ECS, the forcing scenario used has $a_0=\varDelta F$ and $a_i=0$ for all $i>0$, which corresponds to the following step-function :

A3 $$\begin{aligned} F_{step}(t)= \varDelta F \varTheta (t) \end{aligned}$$

where $\varDelta F$ corresponds to a doubling in $\hbox {CO}_{2}$ and $\varTheta (t)$ is again the Heaviside step function. We can calculate the temperature change for this step-increase in forcing at any time following the increase:

A4 $$\begin{aligned} \varDelta T_{step}(t)=-s \varDelta F \left[ \frac{(\tau +t)^{H}-{\tau }^{H}}{\tau ^{H}} \right] \end{aligned}$$

which can be rewritten as:

A5 $$\begin{aligned} \varDelta T_{step}(t)= s \varDelta F \left[ 1 - \left( \frac{t}{\tau } +1 \right) ^H \right] \end{aligned}$$

To obtain the ECS, we only need to take $t \rightarrow \infty$:

A6 $$\begin{aligned} ECS=\varDelta T_{eq}=\varDelta T_{step}(t \rightarrow \infty )= {\left\{ \begin{array}{ll} s \varDelta F &{} H < 0 \\ \infty &{} 0 \ge H \end{array}\right. } \end{aligned}$$

ECS is thus the temperature change at infinity when a steady-state is reached; in this model this requires $H<0$. If $H \ge 0$, we effectively have a runaway greenhouse model as the temperature keeps increasing monotonically. As the scaling exponent approaches 0 from the left, $H \rightarrow 0^-$, the convergence time increases and then diverges for a positive exponent. We also define ECS for a more concrete horizon of 500 years :

A7 $$\begin{aligned} ECS_{500}=\varDelta T_{step}(500 \, years) \end{aligned}$$

This allows us to calculate the warming fraction left for a given scaling exponent *H* between half a millennium and infinity.

To calculate TCR, a different forcing scenario is used where a doubling of carbon dioxide takes place over 70 years, increasing at a rate of $1 \%$ per year, and then held constant. The forcing produced by carbon dioxide is proportional to the logarithm of its concentration and therefore, this exponentially increasing carbon dioxide scenario corresponds to a linear increase in forcing with $a_1=\varDelta F \tau _{tr}^{-1}$ and $a_i=0$ $\forall i \ne 1$ :

A8 $$\begin{aligned} F_{tr}(t)= {\left\{ \begin{array}{ll} 0 &{} t< 0 \\ \varDelta F \frac{t}{\tau _{tr}} &{} 0<t< \tau _{tr} \\ \varDelta F &{} \tau _{tr} <t \end{array}\right. } \end{aligned}$$

where $\tau _{tr}$ is the time over which the doubling takes place, that is 70 years, and $\varDelta F$ is the final change in forcing. We can again calculate the temperature response analytically with the SCRF :

A9 $$\begin{aligned} \varDelta T_{tr}(t)= {\left\{ \begin{array}{ll} \frac{-s \varDelta F}{\tau ^{H} \tau _{tr} (H+1)}\left[ (\tau +t)^{H+1} - {\tau }^{H}(\tau +t(H+1)) \right] &{} 0 \le t \le \tau _{tr} \\ \frac{-s \varDelta F}{\tau ^{H } }\left[ \frac{(\tau +t)^{H+1} - (\tau +t-\tau _{tr})^{H}(\tau +t+\tau _{tr} H))}{\tau _{tr} (H +1)} + (\tau +t -\tau _{tr})^{H} - {\tau }^{H} ) \right] &{} \tau _{tr} \le t \\ \end{array}\right. } \end{aligned}$$

The temperature change at $\varDelta T _{tr}(t=\tau _{tr})$, that is when the forcing reaches its final value, is then defined as the TCR. This measure is not representative of the ECS, although they become equal for sufficiently large negative values of the scaling exponent *H*, but it is even more relevant for near-term projections as the TCR will be the main contribution to global warming as long as the exponential rise in carbon dioxide concentration continues. If we take $t\rightarrow \infty$, we notice that the left term in the bracket vanishes and we are left with Eq. A6. We thus see that the steady-state response does not depend on the forcing scenario as $\varDelta T _{tr}(t \rightarrow \infty )= \varDelta T _{step}(t \rightarrow \infty )$, but only on the change in forcing. It is convenient to analytically calculate the ratio of TCR to ECS as a function of *H* and $\tau$ :

A10 $$\begin{aligned} \frac{TCR}{ECS}=\frac{T_{tr}(\tau _{tr})}{T_{step}(t \rightarrow \infty )} \end{aligned}$$

This ratio is shown on Fig. 2 for $\tau = 2 \, years$ with equilibrium defined at infinity and at 500 years. It decreases monotonically to zero for the former as the scaling exponent approaches zero from the left and the ECS diverges. The gap between TCR and ECS therefore deepens for higher values of H, which explains the sensitivity of ECS to H since the observed warming, similar to the TCR, is a smaller fraction of the ECS. On the other hand, for increasingly negative values of H, the TCR to ECS goes to one as the memory of the model becomes infinitely small, effectively becoming a Dirac $\delta$ function. Also shown, the warming fraction left after 500 years of constant forcing informs us about the longest component of the memory of the model, and that therefore, for values of $H \approx -0.5$ and greater, a sizeable fraction of the warming will occur beyond that horizon.

## Appendix: inner scale of the linear response

In this section, we present an argument in favour of the inner scale of the linear response at approximately 2 years. We mentioned that the physical mechanism which we approximate by integrating the radiative forcing history with the SCRF is the accumulation of heat, mostly, in the oceans which in turn drives the forced temperature response in the atmosphere and at the sea-surface. Therefore, we expect a strong correlation between the two at large time scales where the forced variability overwhelms the internal one, and thus a similar large scale scaling behaviour which should be dominated by the anthropogenic effects. It may seem surprising that up until now, the fundamental issue of land-ocean coupling has, to our knowledge, not been investigated empirically. The likely reason is that it requires the use of Haar, or other appropriately defined, fluctuations. The Haar fluctuation over an interval $\varDelta t$ is simply the difference between the mean over the first and second half of the interval. It is a convenient way to characterize variability as a function of time scale in real space and it is valid whether fluctuations increase or decrease with scale, which is necessary here in order to avoid spurious statistical issues at low-frequencies (Lovejoy and Schertzer 2012)

Figure 11 compares the mean Haar fluctuations as a function of timescale of the global sea-surface temperature (SST), the land-surface air temperature (LST) and the global combined land-ocean surface air temperature (SAT). The forced component of variability dominates for large timescales and we see that SST, LST and SAT have similar forced variability, higher for LST and weaker for SST as expected, and a scaling behaviour akin to the forcing for time scales above 20 years. For short timescales, the internal variability dominates and this leads to diverging behaviour between the SST, LST and the SAT since the turbulent dynamics of the ocean and the atmosphere are different. The driving energy rate density, the turbulent $\epsilon$ that determines the weather-macroweather transition scale $\tau _w$, is about $10^{-3} \,\,\mathrm{W Kg}^{-1}$ in the atmosphere, but only about $10^{-8} \,\,\mathrm{W Kg}^{-1}$ in the ocean surface layer (Lovejoy and Schertzer 2010). The transition time scale is proportional to $\epsilon ^{-\frac{1}{3}}$ so that the weather-macroweather transition in the SST is visible around 2 years rather than at a sub-monthly scale in the atmosphere, about 10 days.

The SAT should scale similarly to LST in the macroweather regime since they are both atmospheric fields with similar turbulent dynamics, but we observe a discrepancy on Fig. 11 : as expected the monthly LST series do not show the sub-monthly transition, but the scaling of SAT below 2 years shows a flattening. It is possible this is just a bias resulting from the inclusion of SST as air-surface temperature in global SAT, which could artificially decrease the high-frequency variability of SAT, but more investigation needs to be done to confirm the claim.

The model presented is not expected to reproduce the internal variability, which is treated as an independant stochastic process, and the high-frequency cutoff $\tau$ was introduced as the smallest scale over which the linear response approximation is expected to hold. As mentioned above, this should translate in correlation between the ocean and the atmosphere forced responses. Figure 12 shows the mean pairwise correlation between the first-order Haar fluctuations of SST, LST and SAT at different timescales. We observe a steep increase to high correlation between all pairs up to 2 years; the SAT series are composed from the LST and SST series and as such it is not surprising to find high correlation, but the completely independent LST and SST series confirm the same transition. This supports our interpretation that as we consider larger time scales, the internal variability averages out and the air surface temperature becomes proportional to the ocean temperature as a consequence of the low-frequency dominance of the forced response from integrated radiative forcing on both. This motivates us to fix the model parameter $\tau = 2$ years for simplicity.

Result for ECS and $\hbox {ECS}_{500}$ are calculated for $\tau \in \{ 1,2,3 \} \, years$ in order to evaluate the sensitivity of the result to this choice (Table 4). We also give an average of the three results weigthed by their relative likelihood (which yields weights of 0.38,0.33 and 0.29 respectively), and the result is in fact very close to the $\tau = 2 \,$ years value used. The likelihood function therefore varies slowly along the direction of $\tau$, with the 3 values almost equally probable, and does not allow to constrain the parameter well by itself. The parameter estimation with a smaller $\tau$ yields an *H* closer to zero, and therefore the high ECS tail thickens as ECS increases as $H \rightarrow 0^-$, and as ultimately diverges for $H \ge 0$. However, the convergence time also increases as $H \rightarrow 0^-$ and so the larger part of the response will happen at multi-centennial, multi-millenial and even much longer timescales. Therefore, the choice of $\tau$ can have a sizable impact for the theoretical ECS at infinity, but the effect will be marginal for any centennial projections and quasi-equilibrium climate sensitivity such as $ECS_{500}$.

**Table 4:** The ECS and $\hbox {ECS}_{500}$ are calculated for different values of the inner scale $\tau$

$\tau$	Median ECS (K)	ECS likely range (K)	Median $\hbox {ECS}_{500}$ (K)	Likely range $\hbox {ECS}_{500}$ (K)
1 year	3.4	[2.2,7.6]	2.3	[1.9,2.8]
2 years	2.3	[1.9,3.4]	2.1	[1.8,2.6]
4 years	2.0	[1.7,2.4]	1.9	[1.7,2.3]
Combined	2.4	[1.8,4.4]	2.1	[1.8,2.6]

A combination of the three results weigthed by their likelihood is also shown

## Appendix: Numerical estimation of the SCRF parameters

This appendix provides extra information concerning the SCRF model parameter estimation technique and tests the efficacy of the method on synthetic time series.

The parameters for the fractional Gaussian noise (fGn) (which is used as a model for the internal variability) are estimated with an iterative procedure. A first estimate is made from the internal variability obtained from a likely combination of parameters; it is then used as the error model to find the parameter combination with maximum likelihood whose associated internal variability serves to obtain the second estimate of the fGn parameters; the maximization process is repeated until the parameters converged, which occurred after only 2 steps. Figure 13 shows the internal variability from the Cowtan & Way (C&W) instrumental temperature series and three other realizations of the same estimated fGn process (Table 5). For CMIP5 GCMs, it would be simpler since the fGn parameters could be estimated directly from unforced control runs which correspond to realizations of the internal variability in each GCM. The following *Mathematica 10.4* (Wolfram Research Inc 2016) functions were used to perform the above calculations : LogLikelihood[proc,data], FractionalGaussianNoiseProcess[$\mu$,$\sigma$,*h*] and EstimatedProcess[data,proc]. Note that the Hurst exponent *h* used within *Mathematica 10.4* describes the scaling behaviour of the associated fractional Brownian motion obtained by integrating the fGn. The notation $H'=h-1$ corresponds to the associated parameter in Lovejoy et al. (2015) which directly describes the scaling associated with the fluctuations of the fGn itself. $H'$ should not be confused with the scaling exponent* H* used to produce the forced temperature response using the SCRF.

**Table 5:** Parameters for the fractional Gaussian noise process used for the error model when calculating the likelihood function for each dataset

Dataset	$\sigma$	$H'$
HadCRUT4	0.10	$-0.24$
C&W	0.10	$-0.26$
GISTEMP	0.12	$-0.17$
NOAAGlobalTemp	0.12	$-0.15$
BEST	0.11	$-0.23$

The mean $\mu$ was set to zero, so only the volatility $\sigma$ and exponent $H'$ are given

To show the efficacy of our method, we test it on simulations generated by adding realizations of fGn, with $\sigma =0.1$ and $H'=-0.25$, onto forced temperature response generated with Eq. 20. Given that it is computationally heavy to calculate the likelihood of a fGn, we restrict the test simulations to $\alpha =1$, $s =1$, $H \in \{-0.2 , -0.6 \}$ and $\nu \in \{ 0.5 , 0.8 \}$. This yields 4 parameter combinations to which are added 25 realizations of fGn with known parameters $(\mu ,\sigma ,h)=(0,0.1,0.75)$, shown on Fig. 14.

Figure 15 shows the resulting PDF for *H* and $\nu$ with $\alpha$ known and the best $s$ found. The likelihood function was calculated $\forall H \in [-1.1,0.3]$ and $\forall \nu \in [0.2,1.0]$ both in increments of 0.05. For each combination of *H* and $\nu$, the best $s$ is found; first an estimate $s_0$ is made by least-square regression and then a maximum likelihood estimate is made $\forall s \in [0.85 {s }_0,1.15 {s }_0]$ in increments of $0.05 {s }_0$. The likelihood function is then interpolated to find the best $s$ and its associated PDF using Bayes’ rule with a uniform prior distribution; the relative error $\delta _s$ for each $s$, defined as $\delta _{s }=\sigma _{s } s^{-1}$ where $\sigma _{s}$ is the one standard deviation defined from the PDF, was found to be very small and consistent between the different parameter combinations $(H,\nu )$. Results are summarized for each set of simulations considered in Table 6.

Therefore, we can recover the parameters accurately with this Bayesian approach with a fGn error model; the 90% confidence intervals are effective, but they remain large and widely varying for single realizations because of the stochastic internal variability.

Decreasing the resolution in *H* to 0.1 does not alter the result significantly and for the parameter estimation from observational data it will be sufficient to calculate the likelihood function $\forall s \in [0.085 {s }_0,1.15 {s }_0]$,$\forall H \in [-1.5,0.2]$ in increments of 0.1, $\forall \nu \in [0.2,1.0]$ in increments of 0.05 and $\forall \alpha \in [-0.3,2.]$ in increments of 0.1. Since the relative error on $s$ is small and consistent, we neglect the uncertainty on this parameter for simplicity and only consider the best value for each *H*, $\alpha$ and $\nu$ parameter combination. The resulting 3-dimensional likelihood surface was then interpolated to higher resolution with a second-order interpolation and used to calculate the probabilities of the climate sensitivities and projections corresponding to each parameter combination.

**Table 6:** Summary of results for test simulations of four $(H,\nu )$ parameter combinations. The median *H* and $\nu$ given along with the associated confidence intervals are obtained from the combination of the PDFs of the 25 individual realizations of fGn

*H*	$\nu$	Median *H*	$90 \%$ CI	$\%$ correct	Median $\nu$	$90 \%$ CI	$\%$ correct	$\delta _s$
$-0.2$	0.8	$-0.18$	[$-0.22$, $-0.14$]	96 $\%$	0.80	[0.78,0.82]	92 $\%$	$0.23 \pm 0.01$
$-0.6$	0.5	$-0.57$	[$-0.68$, $-0.47$]	96 $\%$	0.49	[0.47,0.52]	88 $\%$	$0.123 \pm 0.006$
$-0.2$	0.5	$-0.19$	[$-0.25$, $-0.13$]	96 $\%$	0.49	[0.47,0.52]	88 $\%$	$0.25 \pm 0.01$
$-0.6$	0.8	$-0.58$	[$-0.65$, $-0.51$]	96 $\%$	0.80	[0.77,0.82]	88 $\%$	$0.113 \pm 0.006$

The $\%$ correct corresponds to the number of realizations for which the true parameter lies in the $90\%$ CI. The average relative error $\delta _s$ for all realizations is given with its one standard deviation
